# Electrostatic interactions influence diazabicyclooctane inhibitor potency against OXA-48-like β-lactamases

**DOI:** 10.1039/d5md00512d

**Published:** 2025-08-08

**Authors:** Joseph F. Hoff, Kirsty E. Goudar, Karina Calvopiña, Michael Beer, Philip Hinchliffe, John M. Shaw, Catherine L. Tooke, Yuiko Takebayashi, Andrew F. Cadzow, Nicholas J. Harmer, Adrian J. Mulholland, Christopher J. Schofield, James Spencer

**Affiliations:** a School of Cellular and Molecular Medicine, University of Bristol Bristol BS8 1TD UK Jim.Spencer@bristol.ac.uk; b The Department of Chemistry and the Ineos Oxford Institute for Antimicrobial Research, Chemistry Research Laboratory, University of Oxford 12 Mansfield Road Oxford OX1 3TA UK; c Centre for Computational Chemistry, School of Chemistry, University of Bristol Bristol BS8 1TS UK; d Department of Life Sciences, University of Bath 4 South, Claverton Down Bath BA2 7AY UK; e Living Systems Institute, University of Exeter Stocker Road Exeter EX4 4QD UK

## Abstract

Carbapenemases, β-lactamases hydrolysing carbapenem antibiotics, challenge the treatment of multi-drug resistant bacteria. The OXA-48 carbapenemase is widely disseminated in *Enterobacterales*, necessitating new treatments for producer strains. Diazabicyclooctane (DBO) inhibitors, including avibactam and nacubactam, act on a wide range of enzymes to overcome β-lactamase-mediated resistance. Here we describe investigations on how avibactam and nacubactam inhibit OXA-48 and two variants, OXA-163 and OXA-405, with deletions in the β5–β6 loop neighbouring the active site that modify activity towards different β-lactam classes. Nacubactam is ∼80-fold less potent than avibactam towards OXA-48, but this difference reduces in OXA-163 and OXA-405. Crystal structures and molecular dynamics simulations reveal electrostatic repulsion between Arg214 on the OXA-48 β5–β6 active-site loop and nacubactam, but not avibactam; effects absent from simulations of OXA-163 and OXA-405, which lack Arg214. Crystallographic and mass spectrometry data demonstrate that all three enzymes support desulfation of the bound DBOs. The results indicate that interactions with Arg214 affect DBO potency, suggesting that sequence variation in OXA-48-like β-lactamases affects reactivity towards inhibitors as well as β-lactam substrates.

## Abstract 2

The OXA-48 β-lactamase protects bacteria from carbapenem antibiotics; with activity affected by sequence variation. We show variation to affect susceptibility towards diazabicyclooctane (DBO) inhibitors, dependent upon the inhibitor C2 substituent.

## Graphical abstract

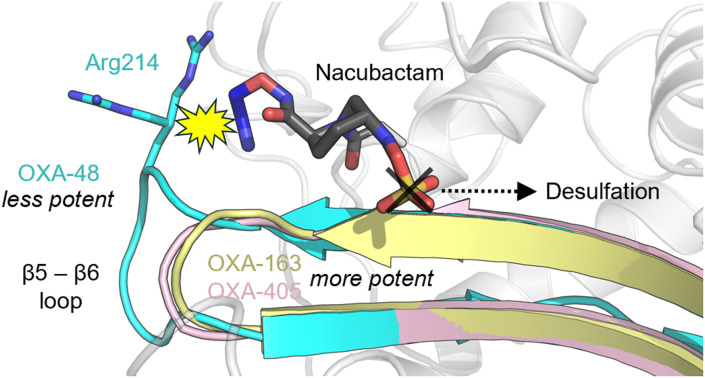

## Introduction

**Fig. 1:**
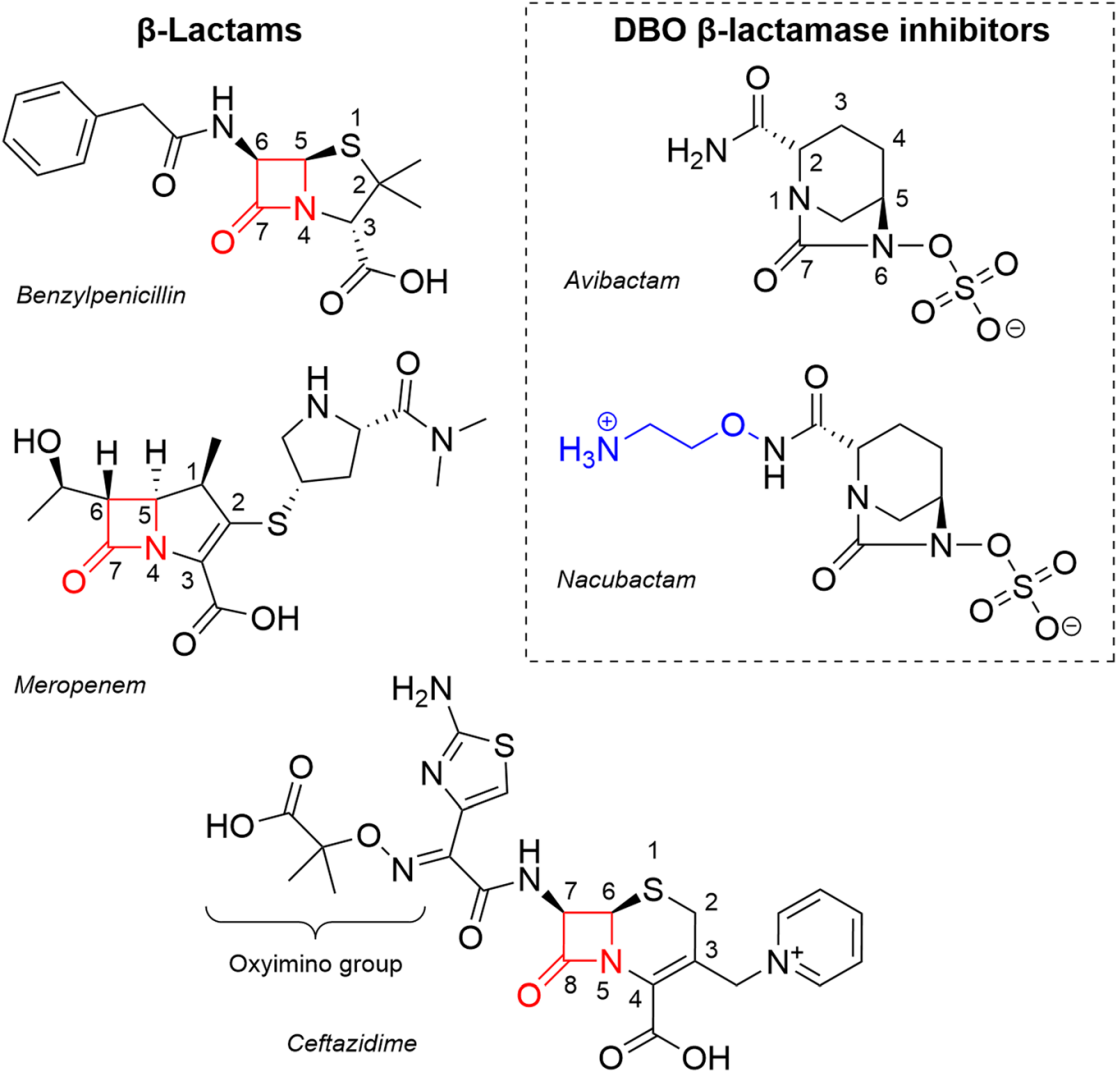
β-Lactam antibiotics and DBO β-lactamase inhibitors. Representative penicillin (benzylpenicillin), carbapenem (meropenem) and cephalosporin (ceftazidime) β-lactam antibiotics, with the β-lactam ring highlighted in red. DBO BLIs used in this work are shown on the right, with the additional 1-aminoethoxy group of nacubactam highlighted in blue. The core ring atoms of avibactam and the β-lactams are numbered.

Antimicrobial resistance (AMR) is a major and increasing threat to global public health, with ∼8.2 million associated annual deaths predicted by 2050.^1^ The β-lactams (penicillins and related agents) are the most commonly prescribed antibiotic class.^2,3^ In Gram-negative bacteria, the dominant β-lactam resistance mechanism is production of β-lactamases^4^ that hydrolyse the four-membered β-lactam ring (Fig. 1), so rendering β-lactams inactive towards their cellular penicillin-binding protein (PBP) targets. β-Lactamases are divided, on the basis of sequence and structure, into four mechanistically distinct classes (A to D) of which classes A, C and D are serine β-lactamases (SBLs) and class B zinc-dependent metallo-β-lactamases (MBLs).^5^ OXA-48 is a class D SBL that uses a nucleophilic serine (Ser70) to hydrolyse β-lactams, with a proximal post-translationally carbamylated lysine (Lys73) proposed to act as the general base for both the acylation and deacylation steps of the reaction.^6^

OXA-48 is of particular clinical importance due to its prevalence in the *Enterobacterales* order of bacteria, which includes two (*Escherichia coli* and *Klebsiella pneumoniae*) of the three pathogens most commonly associated with global AMR-related deaths in 2019.^7^ Worryingly, OXA-48 production causes failure of carbapenems, the class of β-lactam antibiotics often reserved for the most serious bacterial infections. Due to its widespread dissemination, OXA-48 is regarded as one of the five most important acquired β-lactamases responsible for carbapenem resistance.^8,9^ Carbapenem resistant *Enterobacterales* are classed by the World Health Organization as Critical Priority pathogens for global public health.^10^

To circumvent β-lactamase-mediated antibiotic resistance, β-lactamase inhibitors (BLIs) have been developed for co-administration with β-lactam antibiotics.^4^ The diazabicyclooctanes (DBOs) are one group of BLIs that have proven successful in the clinic, with avibactam currently used in combination with the expanded-spectrum oxyiminocephalosporin ceftazidime to treat multi-drug resistant bacterial infections (Fig. 1).^11^ DBOs inhibit SBLs through reversible carbamoylation of the nucleophilic serine, and apparently evade hydrolysis, preferring to recyclise to release the intact DBO.^12–14^

DBO inhibitors are of particular interest in the context of OXA-48, which is generally poorly inhibited by more “classical” β-lactam-based inhibitors (tazobactam, clavulanic acid, sulbactam), but more effectively by avibactam.^6,15^ In all currently available crystal structures of OXA-48 complexes, avibactam shows a very similar binding mode, and appears to induce decarbamylation of the catalytic Lys73, even under basic conditions, an observation which has been attributed to the potency of avibactam inhibition towards class D carbapenemases.^6,14,16,17^ Fröhlich *et al.*^11^ demonstrated that resistance to the ceftazidime : avibactam combination could be achieved in the laboratory through just two amino acid changes in the OXA-48 active site, highlighting the potential for changes in susceptibility to DBO combinations to emerge in clinical OXA-48 variants, and the need for continued inhibitor development to guard against such a possibility.

Following the development and introduction of avibactam,^13^ numerous additional DBO inhibitors have been investigated, they mostly differ in the composition of the C2 substituent group on the core DBO scaffold.^18–20^ One such inhibitor, nacubactam, differs from avibactam by the addition of a 1-aminoethoxy group on the C2 group (Fig. 1), and is currently in phase III clinical trials in combination with the β-lactams cefepime or aztreonam for the treatment of complicated urinary tract infections or uncomplicated pyelonephritis (https://ClinicalTrials.gov identifier NCT05887908).^21^ We have investigated activity of nacubactam, in comparison with avibactam, towards OXA-48 and two clinical variants, OXA-163 and OXA-405, that contain four amino acid deletions within their β5–β6 active site loops and show reduced carbapenemase activity but enhanced hydrolysis of ceftazidime and other oxyiminocephalosporins.^22–24^ A combination of *in vitro* inhibition assays, X-ray crystal structures of enzyme : DBO complexes, and molecular dynamics simulations based upon these leads us to conclude that electrostatic repulsion between the positively charged N atom of the nacubactam C2 substituent and the side-chain of Arg214 on the β5–β6 loop reduces potency towards OXA-48, compared to avibactam. These effects are less pronounced in the OXA-163 and OXA-405 variants, which lack Arg214. These data show that the effects of variation between OXA-48-like β-lactamases extend to interactions with DBO inhibitors, as well as classes of β-lactam substrates.^22–24^ Crystal structures and mass spectrometry provide evidence for desulfation of the DBO-derived carbamoyl-enzyme intermediate to form a hydroxylamine-containing fragment, indicating that class D SBLs can support an alternative breakdown pathway for DBOs.

## Results

### DBO interactions with OXA-48 and its β5–β6 loop variants, OXA-163 and OXA-405

In this work, we sought to investigate the effects of DBO inhibitors on naturally occurring OXA-48 variants differing in their activity towards expanded-spectrum oxyiminocephalosporin (*e.g.* ceftazidime) and carbapenem substrates. To understand the effect of β5–β6 loop composition on DBO inhibition, we undertook *in vitro* inhibition experiments challenging OXA-48 and its naturally occurring variants, OXA-163 and OXA-405, with the DBO inhibitors avibactam and nacubactam. Nacubactam is distinguished from avibactam by an extended C2 substituent bearing a 1-aminoethoxy group (Fig. 1). Both OXA-163 and OXA-405 have four amino acid deletions within the β5–β6 loop, including of residue Arg214. OXA-163 also has a single amino acid substitution (Ser212Asp) adjacent to the deleted region.

Compared to avibactam, nacubactam is a weaker inhibitor of OXA-48, with respective IC_50_ values of 0.26 μM and 19.9 μM, indicating a 77-fold difference in inhibition potency (Table 1). Whilst avibactam remains more potent than nacubactam towards both OXA-163 (IC_50_ values 0.20 μM and 1.23 μM, respectively) and OXA-405 (IC_50_ values 0.99 μM and 10.6 μM, respectively) there are notable increases in nacubactam inhibition potency compared to OXA-48 (16.2-fold for OXA-163, 1.9-fold for OXA-405). Consequently, the differences in inhibition potencies between avibactam and nacubactam are reduced for the two variants (6.2- and 10.7-fold for OXA-163 and OXA-405, respectively). Therefore, the extended β5–β6 loop of OXA-48 appears to be deleterious for the inhibition potency of nacubactam, compared to both OXA-163 and OXA-405.

**Table 1:** Inhibitory values (IC_50_) for nacubactam and avibactam against OXA-48-like β-lactamases

Enzyme	IC_50_ values (μM) by inhibitor				Fold difference
	Nacubactam	95% confidence interval	Avibactam	95% confidence interval	
OXA-48	19.91	[15.78, 25.22]	0.26	[0.20, 0.34]	76.6
OXA-163	1.23	[0.96, 1.58]	0.20	[0.16, 0.25]	6.2
OXA-405	10.60	[8.18, 13.72]	0.99	[0.79, 1.25]	10.7

### Crystal structure of OXA-48 : nacubactam carbamoyl-enzyme complex

**Fig. 2:**
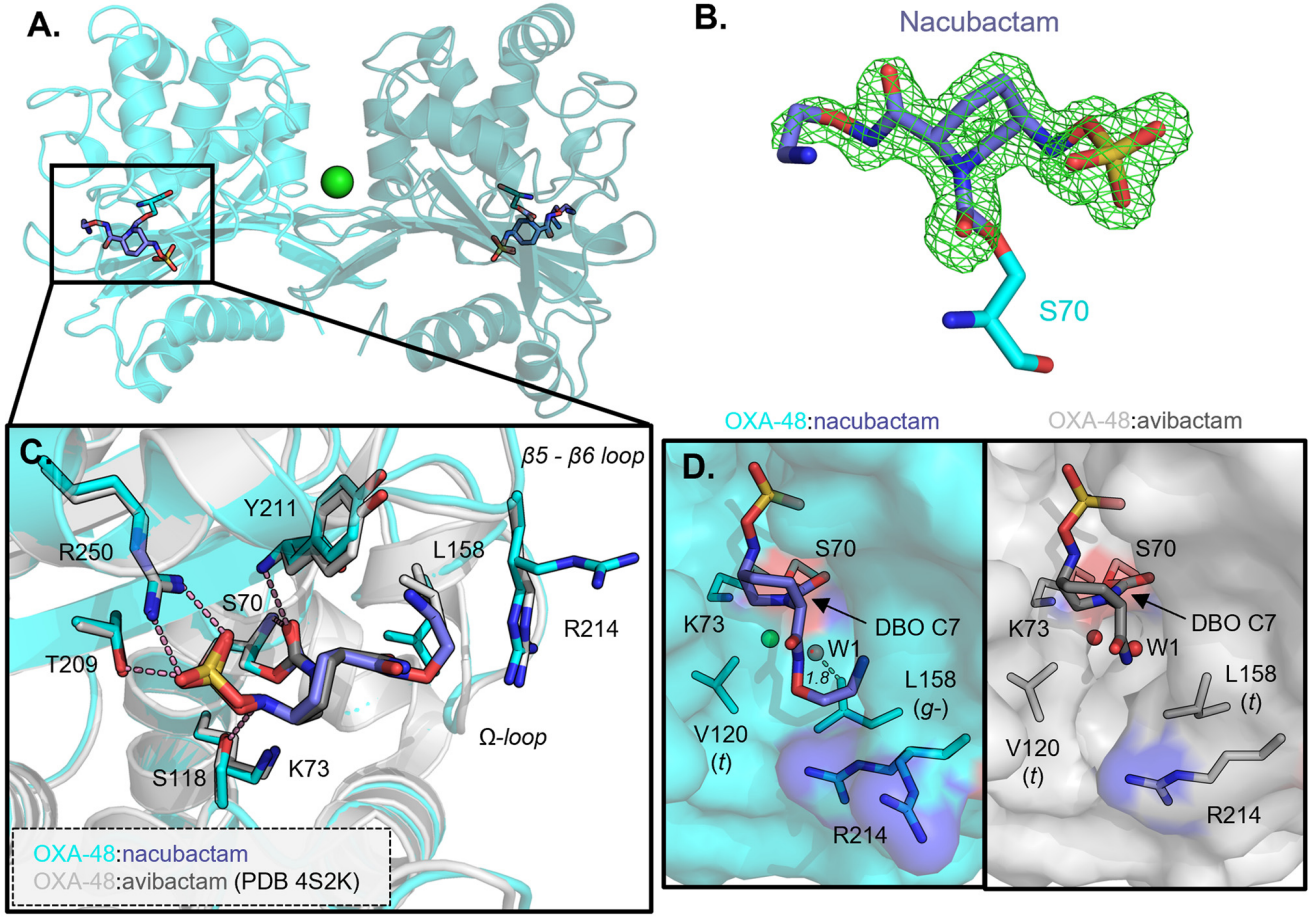
Crystal structure views of OXA-48 bound to nacubactam. (A) Crystal structure of OXA-48 carbamoyl-enzyme complex with nacubactam. Different shades of cyan represent different chains in the homodimer, chloride ion at the dimer interface is shown as a green sphere. (B) Nacubactam-derived carbamoyl-enzyme complex, mesh shows *F*_o_–*F*_c_ electron density from refinement carried out in the absence of ligand, contoured at 3*σ*. (C) Close-up of the chain B active site (cyan), overlaid with a previously determined structure of OXA-48 bound to avibactam (PDB 4S2K,^14^ grey). (D) Surface views of the deacylating water channel in DBO-bound OXA-48 complexes, water molecules and chloride ions are represented as red and green spheres, respectively. Leu158 *χ*_1_ (N-Cα-Cβ-Cγ) and Val120 *χ*_1_ (N-Cα-Cβ-Cγ1) rotamers are highlighted in brackets; dashed line shows distance between the dual-occupancy water molecule (W1) and Leu158 Cδ2.

To investigate the basis for these differences in DBO inhibition potency towards OXA-48 and the two variants in more detail, we sought to determine crystal structures for the respective complexes, comparing as appropriate with the uncomplexed structures. A crystal structure of nacubactam bound to homodimeric OXA-48 was solved at 1.51 Å resolution, from a crystal soaked with nacubactam for 30 min before freezing, with a chloride ion positioned at the dimer interface, as observed previously (Fig. 2A, Table S1A).^25^ Continuous positive *F*_o_–*F*_c_ electron density between the ligand and Ser70 indicates the presence of a covalently bound nacubactam-derived carbamoyl-enzyme complex (Fig. 2B). When overlaid with the structures of uncomplexed OXA-48 (1.38 Å resolution, Table S1A) and OXA-48 bound to avibactam (PDB 4S2K,^14^ 2.10 Å), all three structures have very similar backbones, as highlighted by the low Cα RMSD values (0.41 Å and 0.53 Å for superimposition of the OXA-48 : nacubactam complex with the uncomplexed and avibactam-bound structures, respectively (Fig. S1)). The avibactam and nacubactam carbamoyl-enzyme complexes show a similar binding mode to OXA-48, with the nacubactam C7 carbonyl group positioned within the oxyanion hole formed by the backbone amides of residues Ser70 and Tyr211, as seen in β-lactam-derived acyl-enzyme complexes of OXA-48-like enzymes (Fig. 2C).^6^ Both complexes also show the DBO sulfate group pointing towards the side chains of residues Arg250 and Thr209, with the side chain hydroxyl of Ser118 within hydrogen bonding distance of the DBO N6. Thus, binding of the DBO core is facilitated by complementary electrostatic and hydrogen-bonding interactions, whilst the respective C2 substituents point out of the active site towards the OXA-48 β5–β6 (residues 212–219) and Ω- (residues 144–163) loops.

One notable difference between the active site arrangements of these complexes is the carbamylation status of Lys73, which is decarbamylated in both DBO-bound structures (determined at pH values of 8.8 and 7.5 for the nacubactam and avibactam complexes, respectively, Table S2), but remains carbamylated in the uncomplexed enzyme (structure determined at pH 7.5, Fig. S1, Table S2).^14^ An additional difference is in the positioning of the side chain of residue Leu158 in the active-site Ω-loop, which adopts a *g*− *χ*_1_ side-chain rotamer in the OXA-48 : avibactam complex, whereas the *t* rotamer is observed in uncomplexed and nacubactam-bound OXA-48 (Fig. 2C). It is likely that Leu158 adopts this conformation to avoid steric clashes with the bulkier C2 substituent of nacubactam, compared to avibactam. As Leu158 is part of the so-called ‘deacylating water-channel’, differences are observed in solvent access to the hydrophobic pocket in which Lys73 resides (Fig. 2D).^6,26,27^ Surface views suggest that when Leu158 is in the *t*-rotamer, as in the avibactam complex, this channel is open, as evidenced by presence of a water molecule (W1) proximal to the C7 carbonyl of the carbamoyl-enzyme complex. In contrast, when Leu158 adopts the *g*-rotamer, observed when nacubactam is bound, the channel is closed, and the adjacent water is modelled in dual occupancy with the Leu158 side-chain, due we infer to steric clashes (Leu158 Cδ2 is ∼1.8 Å away from this water molecule).

When OXA-48 is bound to nacubactam, in the active site of chain B, Arg214 of the Ω-loop adopts two conformations. In both conformations, the Arg214 side chain is poorly resolved in the final 2*F*_o_–*F*_c_ electron density map (Fig. S2). Thus, it is possible that the side chain of Arg214 and the C2 tail nitrogen of nacubactam are electrostatically repelled *in crystallo* due to clashing positive charges. Chain A of the nacubactam complex, in contrast, does not show this effect, most likely due to a crystal packing contact, whereby residue Glu132 from an adjacent molecule in a different asymmetric unit can electrostatically interact with Arg214, thus holding it in place. Consequently, the C2 tail group of nacubactam adopts a dual conformation in the chain A active site, presumably as a result of electrostatic repulsion by the Arg214 side chain. Of note, in these experiments OXA-48 was purified and crystallised under basic conditions (purification buffer pH 8.4, crystallisation solution pH 8.8) but it is very likely that both Arg214 and the nitrogen atom of the nacubactam C2 substituent are protonated at physiological pH. Indeed, the predicted p*K*_a_ of Arg214 is 12.2 (PropKa webserver^28^) and that of the nacubactam C2 aminoethoxy nitrogen 8.7–9.1 (AIMNet2/MolGpka webservers,^29,30^ Fig. S3).

### MM MD simulations of DBO-derived carbamoyl-enzyme complexes

**Fig. 3:**
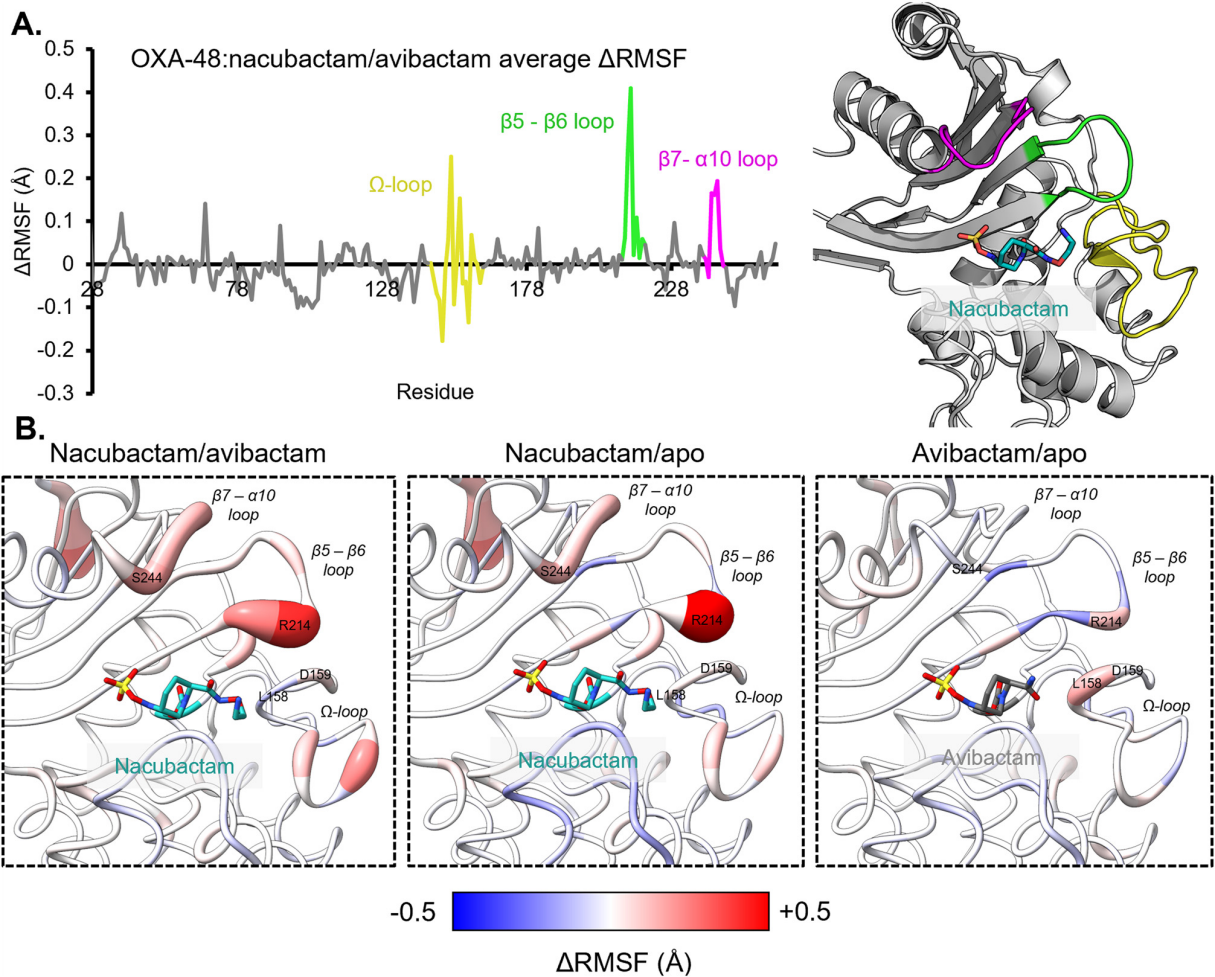
RMSF analysis of MM MD simulations of OXA-48 : DBO carbamoyl-enzyme complexes. (A) Difference between average residue RMSF (ΔRMSF) in simulations of OXA-48 : nacubactam and OXA-48 : avibactam complexes, averaged across both chains of the protein homodimer, plotted against sequence. More positive ΔRMSF values indicate greater fluctuation in nacubactam-, compared to avibactam-bound, OXA-48. The OXA-48 Ω- (yellow, residues 144–163), β5–β6 (green, residues 212–219) and β7–α10 (pink, residues 240–247) loops are highlighted on the graph and OXA-48 : nacubactam crystal structure. (B) Comparisons of ΔRMSF values, coloured according to scale below, between DBO-bound and uncomplexed OXA-48 simulations, mapped onto the crystal structures of OXA-48 bound to nacubactam and avibactam. Thicker and more red backbones indicate more positive ΔRMSF values.

To investigate the possibility that the differences between avibactam and nacubactam binding affect the dynamic behaviour of OXA-48, molecular mechanics molecular dynamics (MM MD) was used to simulate uncomplexed, avibactam- and nacubactam-bound OXA-48. RMSD plots indicate that all simulations were stable over total trajectories of 1.5 μs (Fig. S4). Pairwise differences in average root mean-square fluctuation (ΔRMSF) for each residue, across both active sites of the OXA-48 dimer, were then calculated between the different simulations (Fig. 3). When comparing simulations of the two DBO-bound complexes, three regions of OXA-48, *i.e.* the Ω- (residues 144–163), β5–β6 (212–219) and β7–α10 (240–247) loops, show noticeable changes in ΔRMSF (Fig. 3A). When mapped onto the structure of OXA-48, these regions centre around Arg214, which has an average ΔRMSF of +0.41 Å. This indicates an increase in mobility of this residue in the nacubactam complex, compared to the avibactam complex, consistent with our crystallographic evidence (above) of electrostatic clashes between the nacubactam C2 substituent and the β5–β6 loop (Fig. 2C). Indeed, measuring the distance between Arg214 (Cζ) and the C2 amide nitrogen atoms of each DBO inhibitor over the respective simulation trajectories reveals that this distance is generally longer, with Arg214 adopting a more variable position, in the nacubactam-, compared to the avibactam-bound, complex (Fig. S5). Simulations of the OXA-48 : nacubactam complex, in which the C2 aminoethoxy nitrogen was deprotonated (Fig. S4 and S5), do not show the same degree of repulsion of Arg214, suggesting that the observed displacement is largely due to electrostatic effects.

Arg214 displacement by nacubactam results in an increase in flexibility across adjacent loops in the OXA-48 active site, most notably within the β5–β6 loop itself and, by disruption of a salt bridge with Asp159, across the Ω-loop (Fig. 3). This is highlighted by a reduction in hydrogen bonding between Arg214 (Nη1) and Asp159 (Oδ1 and Oδ2) side-chain atoms when nacubactam, compared to avibactam, is bound to OXA-48 (Fig. S6). The β7–α10 loop, which does not directly interact with bound DBO inhibitors in carbamoyl-enzyme complexes, appears to be destabilised by increased movement of the β5–β6 loop. When compared to simulations of uncomplexed OXA-48, the nacubactam-bound complex shows similar RMSF differences to those obtained from comparison with the avibactam complex. In contrast, such differences are less prominent when the avibactam-bound structure is compared with that of uncomplexed OXA-48, although the Arg214 RMSF increases slightly in the former. However, this does not noticeably affect the stability of neighbouring loops in the same way as was observed for nacubactam-bound, compared to uncomplexed, OXA-48.

In simulations of the OXA-48 : nacubactam complex Leu158, located on the Ω-loop, predominantly adopts the *g*− *χ*_1_ rotamer, as observed in the crystal structure (Fig. S7A). In contrast, in the avibactam-bound or uncomplexed structures the *t*-rotamer is preferred. Conformation of the Leu158 side-chain is therefore specifically limited in the nacubactam-bound complex, as a result of steric restrictions imposed by its larger C2 substituent, compared to that of avibactam, as observed *in crystallo* (Fig. 2D). Val120, the other residue contributing to the OXA-48 deacylating water channel, consistently adopts the *t*-rotamer, as observed in the crystal structures, in all simulations of the uncomplexed and DBO-bound enzyme (Fig. S7B).

### DBO complexes with OXA-48 variants OXA-163 and OXA-405

**Fig. 4:**
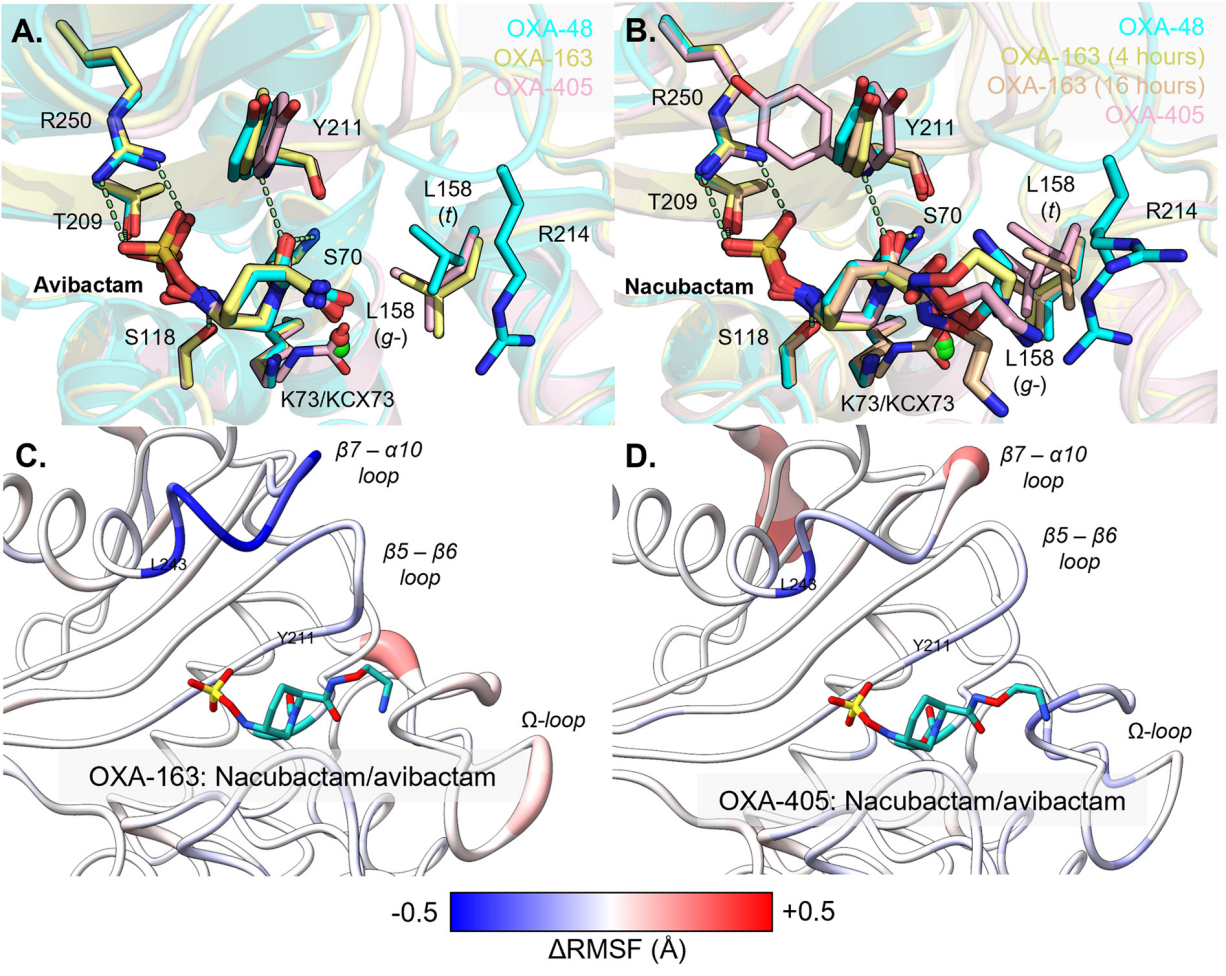
Nacubactam and avibactam inhibition of naturally occurring OXA-48 variants OXA-163 and OXA-405. (A) and (B) Chain B active site overlays of OXA-48 (turquoise), OXA-163 (yellow/orange) and OXA-405 (pink) bound to (A) avibactam and (B) nacubactam. Residues are shown as sticks and numbered according to OXA-48, hydrogen bonding interactions in OXA-48 : DBO structures as dashed lines, chloride ions as green spheres and waters as red spheres. Leu158 *χ*_1_ rotamer form is shown in brackets. (C) and (D) ΔRMSF analysis of MM MD simulations of (C) OXA-163 and (D) OXA-405 complexes with nacubactam and avibactam, mapped onto crystal structures of the respective nacubactam complexes.

Crystal structures were obtained for OXA-163 and OXA-405, as uncomplexed enzymes and in the nacubactam- and avibactam-bound forms (Tables S1B and C). Both OXA-163 and OXA-405 assemble as homodimers in the asymmetric unit. Consistent with their high overall sequence identities, they adopt very similar overall folds and active site arrangements compared to OXA-48 (Fig. S8A and B). The exception is the Ser70 nucleophile, which is in dual conformations in one of the two chains in the crystallographic dimers of uncomplexed OXA-163 and OXA-405. The respective protein backbones only differ at the β5–β6 loop, which is truncated in the cases of OXA-163 and OXA-405, compared to OXA-48 (Fig. S8C). Part of the OXA-405 β7–α10 loop, which neighbours the β5–β6 loop, could not be modelled due to weak electron density, indicating that this loop is more mobile in uncomplexed OXA-405, compared to the other enzymes. β7–α10 loop flexibility coincides with the presence of an alternative side chain rotamer for Tyr211 in the OXA-405:nacubactam complex (Fig. S9). Lys73 is fully decarbamylated in all DBO-bound structures except the OXA-405 : avibactam (both chains), OXA-163 : avibactam (only in chain A) and 16 hour OXA-163 : nacubactam (both chains) complexes, where Lys73 is modelled as partially carbamylated (Table S3), in dual-occupancy with a free lysine side-chain that is associated with either a water molecule or chloride ion (Fig. 4A and B).

In the carbamoyl-enzyme complexes with OXA-405 and OXA-163, both nacubactam and avibactam adopt similar binding modes to those observed with OXA-48 (Fig. 4A and B). These are characterised by complementary electrostatic interactions between the DBO sulfate group and Arg250 (OXA-48 numbering) and positioning of their C2 tail groups close to the β5–β6 loop. The C2 substituent of avibactam is identically positioned in all three enzyme complexes, whereas the additional 1-aminoethoxy group of nacubactam appears more variably located across these structures. Indeed, the unbiased electron density for this extended substituent of nacubactam is less well defined compared to that for the C2 amide component common to the two DBOs, suggesting that in all three enzymes the nacubactam C2 substituent is much more flexible than is the case for avibactam (Fig. S10). A hydroxylamine desulfated carbamoyl-enzyme was also modelled in dual occupancy with the intact avibactam- and nacubactam-derived complexes of both OXA-163 and OXA-405 (Table S4). While only the intact product could be confidently modelled in the crystal structure of OXA-163 : nacubactam obtained from a 4 hour soak, the desulfated species was built into the carbamoyl-enzyme electron density in an additional structure of the same complex determined after a 16 hour soaking time-period (Table S1B, Fig. S10).

Leu158 appears to adopt an alternative rotamer (*g*−) when avibactam is bound to OXA-163 (chain B) and OXA-405 (both chains), compared to OXA-48 or chain A of the OXA-163 : avibactam complex where the *t*-rotamer is observed (Fig. 4A and B). In contrast, when nacubactam is bound, Leu158 is in the *g* rotamer for OXA-48 and OXA-163 (4 hour soak), whilst in OXA-163 (16 hour soak) and OXA-405 complexes both rotamers are observed across the two active sites. Thus, the relationship between the conformation of Leu158, the composition of the β5–β6 loop, and susceptibility to DBO inhibition appears to be structurally complex in the context of OXA-48-like enzymes.

### MM MD simulations of intact DBO-bound OXA-163 and OXA-405 complexes

MM MD simulations of uncomplexed, avibactam- and nacubactam-bound OXA-163 and OXA-405 were then run, using the same protocol as for OXA-48. RMSD plots indicate all simulations to be stable over their respective trajectories (Fig. S4). ΔRMSF analysis reveals no noticeable differences in the flexibility of the β5–β6 loop between simulations of avibactam- and nacubactam-bound OXA-163 and OXA-405 (Fig. 4C and D). The Ω-loop appears slightly more flexible in nacubactam-, compared to avibactam-bound, OXA-163, although the difference is less marked than for OXA-48 (Fig. 3B). The β7–α10 loop, in contrast, is generally less mobile in both the nacubactam, compared to the avibactam, complexes.

Ser118 is suggested to play an important role in DBO reactions with SBLs.^14,17^ Proposed reaction mechanisms for class D SBLs suggest that Ser118 can enable deprotonation of the N6 atom of the DBO carbamoyl-enzyme to promote intramolecular recyclisation, and may also contribute to activation of the decarbamoylating water for hydrolysis.^6,12^ The distances between the DBO N6 and Ser118 Oγ atoms were measured during our MM MD simulations, and the percentage of simulation frames where this distance is within an arbitrarily selected 3.5 Å cutoff calculated (Fig. S11). No major differences in the percentage values were observed between the two independently modelled active sites in the various dimeric complexes. However, the simulations revealed that, in complexes of OXA-48, OXA-163 and OXA-405, N6 of nacubactam was more frequently positioned close to the Ser118 side chain than was the case for avibactam. This suggests that protonation of the nacubactam C2 tail group is required for positioning close to Ser118 and can consequently affect the propensity for recyclisation.

### DBO desulfation in OXA-48-like carbamoyl-enzyme complexes

**Fig. 5:**
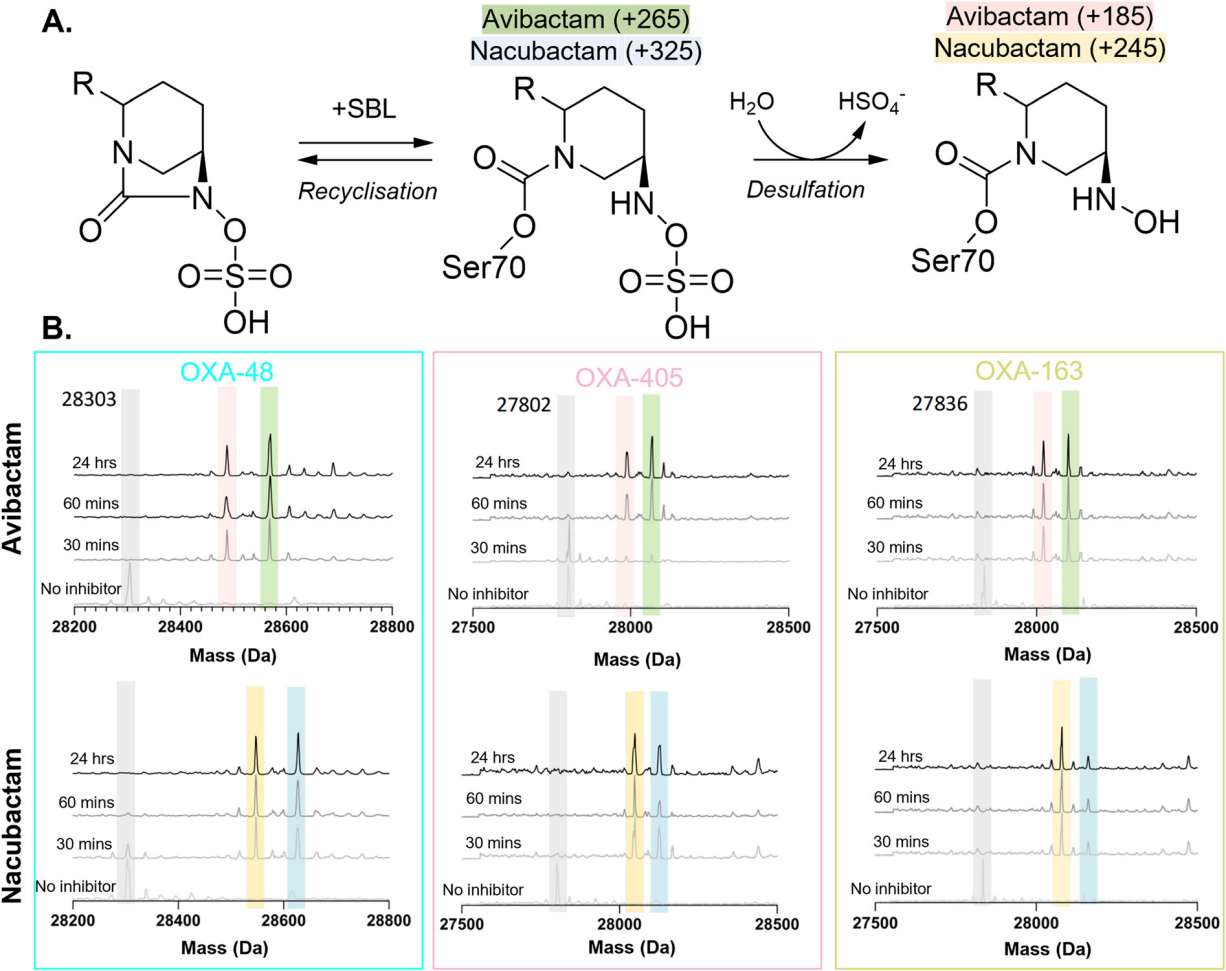
Desulfation of nacubactam- and avibactam-derived carbamoyl-enzyme complexes of OXA-48-like β-lactamases. (A) Structures of proposed DBO-derived carbamoyl-enzyme fragments identified by electrospray ionisation mass spectrometry (ESI-MS). The mass shifts (Da) of the respective nacubactam- and avibactam-derived complexes are shown in brackets. (B) Mass spectra of OXA-48, OXA-405 and OXA-163 as uncomplexed enzymes and following incubation with nacubactam and avibactam at time-points ranging from 30 minutes to 24 hours. Peak colours correspond to species highlighted in panel A.

We also considered the possibility of alternative DBO turnover pathways by OXA-48-like β-lactamases, namely desulfation of the DBO N6 substituent, followed by hydrolysis (Fig. 5A).^31–34^ We used mass spectrometry to monitor time-dependent carbamoylation and fragmentation of nacubactam- and avibactam-derived complexes of OXA-48, OXA-163 and OXA-405 (Fig. 5B). Following 24 hour incubation with both DBOs, for all three enzymes mass shifts associated with the formation of carbamoyl-enzyme complexes with avibactam (+265 Da) and nacubactam (+325 Da) were identified. An additional peak corresponding to an 80 Da loss, relative to the carbamoyl-enzyme, was also observed for both DBOs with these enzymes, suggesting partial desulfation of the DBO carbamoyl-enzyme complexes to form an N6 hydroxylamine-containing fragment. We note that, for all three OXA enzymes, increased desulfation was observed for nacubactam, compared to avibactam.

We also see evidence of desulfation in our crystal structures of OXA-163 and OXA-405 bound to both avibactam and nacubactam, indicated by negative *F*_o_–*F*_c_ difference density around the DBO sulfate group when only the intact carbamoyl-enzyme is modelled (Fig. S12). The desulfated hydroxylamine group can form hydrogen bond networks with the active sites of OXA-163 and OXA-405 *via* a water molecule, observed in a similar position to an equivalent water in structures of the uncomplexed enzymes, that is in dual occupancy with the intact carbamoyl-enzyme sulfate group (Fig. S13). In contrast, we do not see carbamoyl-enzyme desulfation in crystal structures of nacubactam complexes of OXA-48, or of OXA-163 after a shorter (4 hour) soaking time (Fig. S10).

## Discussion

OXA-48 has become one of the most commonly detected carbapenemases in *Enterobacterales* globally, thus treatment options that circumvent β-lactam antibiotic resistance as a result of OXA-48-production are of clinical importance.^8^ DBO-based BLIs are an established option, and various efforts have been made to optimise their activity, with multiple compounds having modified C2 substituents compared to avibactam, the original DBO now available in the clinic.^4^ The degree of modification to the C2 substituent appears to correlate with intrinsic antibacterial, as well as β-lactamase inhibitory, activity resulting from effects upon the ability to bind to both PBPs and SBLs. This gives rise to the possibility of a ‘dual-action’ agent that would not require combination with a β-lactam for treatment of antibiotic-resistant infections.^35,36^ Indeed, nacubactam, which compared to avibactam has an extended 1-aminoethoxy group on its C2 substituent, has been shown to have some antimicrobial activity, which has been attributed to its ability to inhibit PBP2 in *Enterobacterales*.^21^

Our biochemical and structural studies, comparing inhibition of OXA-48 and its naturally occurring variants OXA-163 and OXA-405 by nacubactam and avibactam, have clinically relevant implications for DBO mediated SBL inhibition. Importantly, the IC_50_ values show nacubactam to be a substantially weaker inhibitor of OXA-48 than avibactam, whereas differences in the potencies of the two DBOs decrease in OXA-163 and OXA-405, which both have four amino acid deletions in the β5–β6 active site loop.

We determined high-resolution crystal structures of uncomplexed, avibactam- and nacubactam-bound OXA-48, OXA-163 and OXA-405 to investigate how differences in the β5–β6 loop composition relate to DBO potency. The structural studies reveal conformational variability of this loop only in the OXA-48 : nacubactam complex, as a result of electrostatic repulsion between the C2 tail nitrogen of the nacubactam 1-aminoethoxy group and the side chain of Arg214. Although we do note the possibility of the C2 tail nitrogen deprotonating upon binding, to avoid these electrostatic clashes with Arg214, this would also likely be associated with a thermodynamic penalty.^37^

MM MD simulations of these structures reveal increased flexibility of the β5–β6 loop in nacubactam-bound OXA-48, compared to the uncomplexed or avibactam-bound enzyme, likely due to DBO-mediated electrostatic displacement of Arg214, which consequently appears to propagate flexibility in neighbouring active site loops. This effect has also been shown in a structure of an OXA-48 P68A mutant in complex with the oxyimino-cephalosporin antibiotic ceftazidime (PDB 6Q5F), where the extra bulk of the ceftazidime oxyimino group (Fig. 1) appears to disrupt the interaction between Arg214 and Asp159. The authors suggest that this increases flexibility of the Ω-loop, as evidenced by a lack of electron density for this region in the crystal structure.^11^ Therefore, it appears that Arg214 is an important determinant of OXA-48 active site conformational flexibility, and its displacement may begin to explain the weaker IC_50_ value of nacubactam towards OXA-48, compared to avibactam. Arg214 is considered to contribute to the carbapenemase activity of OXA-48, possibly through its stabilisation of the Ω-loop,^38,39^ modifications to the active site electric field^40^ and/or hydration.^41^ The data presented here additionally support involvement of Arg214 in DBO inhibition of OXA-48-like enzymes.

Note, however, that nacubactam potency is not fully restored to levels observed for avibactam when Arg214 is deleted, as in OXA-163 and OXA-405, suggesting that additional factors can affect DBO potency towards these enzymes. In MM MD simulations of all three enzymes, we see a greater propensity for nacubactam, compared to avibactam, to adopt a binding pose that may more readily facilitate DBO recyclisation, as indicated by the closer proximity of the DBO N6 nitrogen and the Ser118 side-chain oxygen. It is thus possible that, compared to avibactam, nacubactam is more readily recyclised by OXA-48-related enzymes, therefore reducing its inhibitory potency by reducing the lifetime of the carbamoyl-enzyme complex. Indeed, kinetic parameters determined by Outeda-García *et al.*^42^ show an approximately 37-fold decrease in enzyme : inhibitor residence time (*t*_1/2_) and 36-fold increase in dissociation rate (*k*_off_) for nacubactam, compared to avibactam, against OXA-48. These authors also demonstrate that other DBO inhibitors with extended C2 substituents containing ionisable nitrogen atoms (relebactam, zidebactam) have much lower potencies against OXA-48, compared to avibactam (*K*_i_ values of greater than 800 μM *versus* 3.8 μM for avibactam). Another study, on two other DBOs with similarly modified C2 substituents, FPI-1465 and FPI-1602, also showed lower binding affinities and complex residence times against OXA-48 (Fig. S14).^35^ These findings therefore suggest that, in general, such extensions to the DBO C2 substituent are deleterious to inhibitory potency against OXA-48.

We observed desulfation of the DBO N6 substituent by both crystallographically and through mass spectrometry, suggesting that the DBO carbamoyl-enzyme complex with OXA-48-like enzymes can be resolved through more than one pathway. These results extend the observation of DBO desulfation to class D enzymes, showing that desulfation can occur for DBO derived complexes with all SBL classes.^32,33^

We also see differences between the various complexes of OXA-48 family members, in both crystal structures and in simulations, in the position of Leu158 in the deacylating water channel. It appears that steric considerations, with respect to the extended C2 1-aminoethoxy substituent of nacubactam, restrict the flexibility of Leu158, thus resulting in a closed deacylating water channel when OXA-48 is bound to nacubactam. This may affect solvent accessibility of the hydrophobic pocket in which Lys73 resides, in turn influencing the carbamylation status of Lys73 due to the requirement for water access to mediate lysine decarbamylation.^12,43–45^ Such an effect has been observed in time-resolved crystallographic studies of avibactam carbamoylation in the class D SBL CDD-1, that show corresponding movement of Leu158 and release of carbon dioxide following Lys73 decarbamylation.^26^ The consequence, for DBO susceptibility, of Leu158 side-chain position and Lys73 carbamylation status is likely to be complex due to the multiple DBO turnover pathways that can occur in class D SBLs.^12^ The variability of Leu158 rotamers in our crystal structures of DBO complexes with OXA-163 and OXA-405 highlights this.

In conclusion, this work suggests a structural relationship between DBO inhibitory potency and β5–β6 active site loop composition in class D β-lactamases of the OXA-48 family. Our findings thus suggest that, while variants differ in susceptibility to individual DBOs, rational design strategies, involving optimising C2 substituents to make more favourable interactions with the active site, present one route to more potent DBO inhibitors of OXA-48 family SBLs.

## Methods

### Recombinant enzyme expression and purification

For crystallography and kinetic experiments, genes encoding the mature OXA-163^23-261^ (GenBank HQ700343) and OXA-405^23-261^ (GenBank KM589641) open reading frames were codon-optimised and synthesised (Eurofins Genomics), amplified by PCR and cloned into the pOPINF T7 expression vector by recombination (InFusion, Takara) using the primers given in Table S5.^46^*E. coli* BL21-DE3 cells (Novagen) were transformed with pOPINF-OXA-48^(23-265)^ (previously cloned as described in Cahill *et al.*^47^), pOPINF-OXA-163^(23-261)^ and pOPINF-OXA-405^(23-261)^ by heat shock and grown in 2X YT broth supplemented with 50 μg mL^−1^ carbenicillin (Fisher Scientific) for large scale growth (37 °C, 180 rpm shaking). When the culture reached an OD_600_ of 0.6–0.8, expression was induced by the addition of 0.5 mM IPTG (Calibre Scientific) and left overnight at 18 °C, 180 rpm shaking. Cells were then pelleted by centrifugation (6500 × *g*, 10 minutes) and resuspended in 50 mL of the respective purification buffer (50 mM Tris pH 8.4, 0.25 M NaCl, 20% (v/v) glycerol (OXA-48); 150 mM HEPES pH 8.0, 0.4 M NaCl (OXA-163); or 150 mM HEPES pH 6.5, 0.4 M NaCl (OXA-405)) supplemented with 10 mM imidazole, 1 tablet of cOmplete™ EDTA-free protease inhibitor cocktail (Roche, 05056489001), 1 μL benzonase endonuclease (Novagen, 70664) and 2–5 mg lysozyme (Sigma, 62971). All subsequent purification steps were completed at 4 °C or on ice. Cells were lysed using a cell disruptor (Constant Systems) at 25 kpsi and centrifuged at 100 000 × *g* for 1 hour. The soluble fraction was then added to 4 mL Ni-NTA (nitrilotriacetic acid) beads (Qiagen, 1018244) pre-washed in water, and incubated for 1 hour with rotation. The beads were washed once with 10 mL of the respective purification buffer supplemented with 10 mM imidazole, and once more with 10 mL purification buffer supplemented with 20 mM imidazole. Protein was then eluted and collected with the addition of 10 mL of the respective purification buffer supplemented with 300 mM imidazole. NaCl was omitted from the elution step for OXA-163 and OXA-405. The eluent was buffer-exchanged to reduce the imidazole concentration to <50 mM using a 10 kDa cutoff Vivaspin Centrifugal Concentrator (Sartorius, 10738231) and subsequently incubated overnight with 2 mg recombinant 6His-tagged 3C protease to cleave the enzyme purification tag.^48^ The mixture was run through a second 10 mL Ni-NTA column to remove the 3C protease and cleaved hexahistidine tags, and the eluent from this column collected, concentrated to 5 mL and loaded onto a 120 mL HiLoad 16/600 Superdex 75 size-exclusion column (Cytiva, GE28-9893-33) equilibrated with OXA-48 purification buffer, or 0.1 M sodium phosphate buffer at pH 8.0 or pH 6.5 for OXA-163 or OXA-405, respectively. Peak fractions were collected (purity determined by SDS-PAGE^49^), pooled and concentrated to 11.5 mg mL^−1^ (OXA-48), 7.7 mg mL^−1^ (OXA-163) or 11.1 mg mL^−1^ (OXA-405). Enzyme concentration was measured using a NanoDrop spectrophotometer (ThermoFisher) with extinction coefficients calculated using Expasy ProtParam.^50^ Final samples were snap-frozen in liquid nitrogen and stored at −80 °C.

### Ligand soaking and crystal structure determination

The structure of unliganded OXA-48 was determined using diffraction data collected from crystals that grew in 0.1 M HEPES pH 7.5, 33% (v/v) PEG 400, identified from the Morpheus MemGold2 sparse matrix screen (Molecular Dimensions). OXA-48, OXA-163 and OXA-405 crystals used for structure determination of nacubactam-bound complexes were grown in 0.1 M Tris pH 8.8–9.0, 20–50% (v/v) PEG 400 at 10 °C, whilst avibactam-bound and unliganded structures of OXA-163 and OXA-405 were determined from crystals grown in 0.1 M Tris pH 8.5–9.0, 28–32% (v/v) PEG 550 at 19 °C. Crystals were soaked with either 2.5–5 mM nacubactam or 15–100 mM avibactam and either cryoprotected with 20% (v/v) glycerol before freezing, or frozen directly by immersion in liquid nitrogen. See Table S2 for further details on crystallisation and ligand soaking experiments.

X-ray diffraction data were collected at beamlines I03, I04 and I24 of Diamond Light Source (Didcot, UK) or PROXIMA 2A beamline of SOLEIL (Paris, France) (see Table S1 for X-ray collection and refinement statistics). Diffraction images were processed using the in-house Xia2 dials and Xia2 3dii pipelines, or merged *via* AIMLESS (CCP4), ensuring CC_1/2_ greater than 0.3.^51,52^ 5% of reflections in each dataset were reserved to calculate *R*_free_ values. Phases for uncomplexed structures were solved by molecular replacement in PhaserMR (Phenix) using an AlphaFold2 prediction as a search model.^53,54^ Structures were then refined in phenix.refine using their respective uncomplexed structure (with waters removed) as a starting model for one round of rigid body fitting, followed by manual rebuilding and local refitting in Coot.^55,56^ Ligands were fitted into active site *F*_o_–*F*_c_ difference density with ligand geometry restraints generated by Grade2.^57^ Figures of structures were generated in open-source PyMOL 3.0.0 (Schrödinger) or Chimera 1.17.3 (UCSF).^58,59^

### Enzyme kinetics

All enzyme kinetics experiments were performed in 50 mM HEPES pH 7.6, 50 mM NaPO_4_ buffer supplemented with 50 mM NaHCO_3_ and 50 μg mL^−1^ bovine serum albumin (BSA) in 96-well half area plates (Greiner, 675801). Nitrocefin hydrolysis was determined with 1 nM enzyme, measuring changing absorbance at 486 nm over 10 min (Δ*ε* 486 = 20 500 M^−1^ cm^−1^) using a Clariostar plate reader (BMG LabTech).^60^ Steady-state parameters (*K*_M_, *k*_cat_) were calculated in Graphpad Prism v10.2 (Table S6). IC_50_ values were determined as described in Tooke *et al.*,^33^ pre-incubating 1 nM enzyme with differing concentrations of inhibitor diluted in kinetics buffer for 10 minutes before adding 75 μM nitrocefin and measuring change in absorbance at 486 nm over 10 min.

### Mass spectrometry

Avibactam or nacubactam (10 μM) were individually added to OXA-48, OXA-163 or OXA-405 (1 μM) in 50 mM Tris pH 7.5. Queued samples were then analysed with an Acquity-UPLC system (Waters Corporation) coupled to a Xevo G2-S QTof mass spectrometry system (Waters Corporation), using a ProSwift™ RP-4H 1 mm 50 mm column (Thermo Fisher Scientific). Samples were injected from the autosampler onto the column in 95% (v/v) water, 5% (v/v) acetonitrile, 0.1% (v/v) formic acid and eluted using a gradient to 5% (v/v) water, 95% (v/v) acetonitrile, 0.1% (v/v) formic acid, before being introduced into the ESI source. The retention times of all three enzymes proteins were between 5–6 min. Data were analysed using MassLynx 4.1 (Waters Corporation), with deconvolution using the MaxEnt1 algorithm until convergence.

### Molecular dynamics simulations

Extended molecular mechanics/molecular dynamics (MM MD) simulations (1.5 μs) were run for the uncomplexed, nacubactam- and avibactam-bound OXA-48, OXA-163 and OXA-405 structures. As a starting point for MM MD simulations a previously determined structure of OXA-48 bound to avibactam (PDB 4S2K^14^) was re-refined in phenix.refine^55^ with a chloride ion added to the dimer interface following inspection of the *F*_o_–*F*_c_ difference map, replacing the water originally modelled. As two dimers reside in the asymmetric unit of 4S2K, chains A and C were removed. Starting structures for simulations of uncomplexed and nacubactam-bound OXA-405 had sections of the β7–α10 loop that were not modelled in the final crystal structure (residues 237–242) added using Fit Loop (by Rama Search) in Coot.^56^ The 4-hour soak structure of OXA-163 bound to nacubactam was chosen for simulations over the 16-hour structure of the same complex due to a better fit of the modelled ligand in the experimental electron density (Table S4 and Fig. S10). All starting models had crystallographic precipitant molecules removed, excepting the chloride ion found at the dimer interface of all enzyme structures. For the relevant DBO-bound complexes, dual-occupancy carbamylated Lys73 and desulfated carbamoyl-enzymes were removed from starting structures. Amino acid protonation states were altered as predicted by PropKa, based on a system at pH 7.4.^28^ Partial charges for avibactam, nacubactam and the carbamylated active site lysine were calculated using the R.E.D webserver.^61^ Ligand forcefields were generated using general Amber force field (GAFF) parameters and the ff14SB MM forcefield was used for standard protein residues. Hydrogens were added to each complex by tleap (Amber20^62^) and systems solvated within a TIP3P water box^63^ whose edges were at least 10 Å from any protein atoms, with sodium counterions added to balance the overall system charge. All non-water atoms were initially restrained (restraint weight of 100 kcal mol^−1^ A^−2^) for water minimisation (100 cycles of steepest descent followed by 200 cycles of conjugate gradient), followed by minimisation of all hydrogen atoms, water molecules and chloride ions (1000 cycles of steepest descent, 2000 cycles of conjugate gradient). Systems were then heated to 298 K over 20 ps of simulation using a Langevin thermostat, and system pressure was equilibrated to 1 atm over 500 ps with a Berendsen barostat, restraining just the Cα atoms in both steps (restraint weights were 5 kcal mol^−1^ A^−2^). Production simulations were run over 500 ns, repeating three times for a total sampling time of 1.5 μs for each complex. All simulation analyses were performed using CPPTRAJ,^64^ with RMSD values calculated relative to the first deposited simulation structure (at 0.4 ns), excluding all hydrogen atoms and N-terminal residues up to and including Glu24 in both chains of the homodimer.

## Author contributions

J. F. H., K. E. G., K. C., C. J. S. and J. S. conceived the experiments. J. F. H., K. E. G. and A. F. C. performed laboratory experiments and crystallographic data collection/processing, with supervision from C. L. T., P. H., J. M. S., Y. T. and N. J. H. K. C. performed mass spectrometry experiments. J. F. H. undertook molecular simulations with training and supervision from M. B. and A. J. M. J. F. H. and K. E. G. drafted the manuscript, with revisions by J. S. and input from and final approval by all authors.

## Conflicts of interest

There are no conflicts of interest to declare.

## Abbreviations

BLI: β-Lactamase inhibitor
DBO: Diazabicyclooctane
MM MD: Molecular mechanics molecular dynamics
ESI-MS: Electrospray ionisation mass spectrometry
OXA: Oxacillinase
RMSD: Root mean-squared deviation
RMSF: Root mean-squared fluctuation
SBL: Serine β-lactamase

## Supplementary Material

MD-OLF-D5MD00512D-s001

## Data Availability

Supplementary data (sequences of oligonucleotide primers; conditions, data processing and validation statistics for crystallographic experiments; steady-state kinetic parameters for hydrolysis of reporter substrate (nitrocefin); supplementary images of crystal structures; analyses of molecular simulations) are provided in the supplementary information (SI). See DOI: https://doi.org/10.1039/D5MD00512D. Structure factors and coordinates for all crystal structures presented here have been deposited with the Protein Data Bank (PDB) with the following accession numbers: uncomplexed OXA-48 (9H11), OXA-48 in complex with nacubactam (9H12), uncomplexed OXA-163 (9H13), OXA-163 in complex with avibactam (9H14), OXA-163 in complex with nacubactam (4 hour soak) (9H15), OXA-163 in complex with nacubactam (16 hour soak) (9HPV), uncomplexed OXA-405 (9H16), OXA-405 in complex with avibactam (9H17), OXA-405 in complex with nacubactam (9H18). Data from molecular dynamics simulations will be made freely available at the University of Bristol Research Data Repository (https://data.bris.ac.uk/). Analysis scripts will be made available upon request.
