# Supplementary material for: Electrostatic interactions influence diazabicyclooctane inhibitor potency against OXA-48-like β-lactamases
Source: RSC Med Chem. 2025 Aug 8;16(11):5441–55. doi: 10.1039/d5md00512d (PMC12415477; doi:10.1039/d5md00512d)
Supplement: MD-016-D5MD00512D-s001 [file MD-016-D5MD00512D-s001.pdf]

Supplementary Information (SI) for RSC Medicinal Chemistry.
This journal is © The Royal Society of Chemistry 2025

Supplementary Information

Electrostatic interactions influence diazabicyclooctane inhibitor potency against OXA-48-like β-lactamases

Joseph F. Hoff[1†], Kirsty E. Goudar[1†], Karina Calvopiña[2], Michael Beer[1,3], Philip Hinchliffe[1], John M. Shaw[1], Catherine L. Tooke[1,4], Yuiko Takebayashi[1], Andrew F. Cadzow[5], Nicholas J. Harmer[5], Adrian J. Mulholland[3], Christopher J. Schofield[2] and James Spencer[1*]

[1]School of Cellular and Molecular Medicine, University of Bristol, Bristol, United Kingdom, BS8 1TD, U.K.

[2]Chemistry Research Laboratory, Department of Chemistry and the Ineos Oxford Institute of Antimicrobial Research, University of Oxford, 12 Mansfield Road, Oxford, OX1 3TA, U.K.

[3]Centre for Computational Chemistry, School of Chemistry, University of Bristol, United Kingdom, BS8 1TS, U.K.

[4]Department of Life Sciences, 4 South, Claverton Down, University of Bath, BA2 7AY, U.K.

[5]Living Systems Institute, University of Exeter, Stocker Road, Exeter EX4 4QD, U.K.

[†]Equal contributions

*Corresponding author: E-mail: Jim.Spencer@bristol.ac.uk

Supplementary Information

Contents

Table S1A: OXA-48 X-ray data collection and structure refinement statistics

Table S1B: OXA-163 X-ray data collection and structure refinement statistics

Table S1C: OXA-405 X-ray data collection and structure refinement statistics

Table S2: Crystallisation and ligand soaking experiment summary

Table S3: Carbamylated Lys73 (KCX73) fitting statistics.

Table S4: DBO complex ligand fitting statistics

Table S5: Primer sequences used to create OXA-163 and OXA-405 expression constructs

Table S6: Steady state kinetic parameters for nitrocefin hydrolysis by OXA-48, OXA-163 and OXA-405

Figure S1: Overlay of uncomplexed and DBO-bound OXA-48 enzyme crystal structures.

Figure S2: *In crystallo* conformation of Arg214 in uncomplexed and DBO-bound OXA-48

Figure S3: Nacubactam ionisable group predicted pKa values

Figure S4: RMSD plots of MM MD simulation trajectories

Figure S5: Distance analysis of Arg214 relative to avibactam and nacubactam across MD simulation trajectories

Figure S6: Hydrogen bonding analysis between Arg214 and Asp159 over MD simulation trajectories

Figure S7: OXA-48 deacylating water channel rotamer analysis over MD simulations

Figure S8: Overlay of OXA-48, OXA-163 and OXA-405 apoenzyme crystal structures

Figure S9: Conformational flexibility of Tyr211 and β7 – α10 loop in the OXA-405:nacubactam complex.

Figure S10: DBO-derived carbamoyl-enzyme unbiased F_o-F_c omit maps

Figure S11: Analysis of distances between Ser118 Oγ and DBO N6 atoms in simulations of DBO carbamoyl complexes of OXA-48-related enzymes.

Figure S12: Intact DBO-derived carbamoyl-enzyme negative F_o-F_c difference maps.

Figure S13: Water-mediated interactions between the hydroxylamine of desulfated DBO-derived complexes with OXA-405 and OXA-163 active site.

Figure S14: Alternative DBO inhibitors with ionisable C2 substituents.

Table S1A: OXA-48 X-ray data collection and structure refinement statistics. *[a]Experiments were performed at [a]I03, [b]I04, [c]I24 beamlines of the Diamond Light Source (Didcot, UK), or [d]PROXIMA 2A beamline of SOLEIL (Paris, France). Outer shell statistics are in brackets.*

	**Uncomplexed OXA-48[a]**	**OXA48: nacubactam[a]**
**PDB ID**	9H11	9H12
**Data collection**		
Space group	$P6_522$	$P6_522$
Molecules/ASU	2	2
Cell dimensions		
a, b, c (Å)	123.51, 123.51, 161.56	121.76, 121.76, 159.84
α, β, γ (°)	90, 90, 120	90, 90, 120
Wavelength (Å)	0.8622	0.9763
Resolution (Å)	61.76 - 1.38 (1.40 - 1.38)	60.88 - 1.51 (1.54 - 1.51)
R_{pim} (I)	0.055 (1.020)	0.047 (0.956)
$CC_{1/2}$	0.999 (0.309)	0.999 (0.327)
I/σ (I)	8.9 (0.8)	11.3 (0.8)
Completeness (%)	100 (100)	100 (100)
Redundancy	40.2 (41.1)	39.5 (33.5)
**Refinement**		
Resolution (Å)	61.76 - 1.38 (1.43 - 1.38)	60.88 - 1.51 (1.56 - 1.51)
No. reflections	148,214	109,282
R_{work} / R_{free}	0.170 / 0.187	0.173 / 0.192
No. atoms		
Protein	4104	4116
Solvent	688	506
Ligand	-	63
B-factors (Å^2)		
Protein	17.7	20.4
Solvent	30.3	34.6
Ligand	-	20.8
RMS deviations		
Bond angles (°)	0.78	0.84
Bond lengths (Å)	0.0054	0.0064
Ramachandran (%)		
Outliers	0	0
Favoured	97.92	98.56

Table S1B: OXA-163 X-ray data collection and structure refinement statistics. *[a]Experiments were performed at [a]I03, [b]I04, [c]I24 beamlines of the Diamond Light Source (Didcot, UK), or [d]PROXIMA 2A beamline of SOLEIL (Paris, France). Outer shell statistics are in brackets.*

	**Uncomplexed OXA-163[d]**	**OXA-163: avibactam[c]**	**OXA-163: nacubactam (4 hour soak)[a]**	**OXA-163: nacubactam (16 hour soak)[c]**
**PDB ID**	9H13	9H14	9H15	9HPV
**Data collection**				
Space group	$P6_522$	$P6_522$	$P2_12_12$	$P6_522$
Molecules/ASU	2	2	2	2
Cell dimensions				
a, b, c (Å)	122.55, 122.55, 160.43	122.60, 122.60, 160.53	70.62, 79.04, 126.25	122.43, 122.43, 160.47
α, β, γ (°)	90, 90, 120	90, 90, 120	90, 90, 90	90, 90, 120
Wavelength (Å)	0.9000	0.9500	0.9763	0.9500
Resolution (Å)	48.69 - 1.44 (1.46 - 1.44)	106.17 - 1.56 (1.59 - 1.56)	52.66 - 2.18 (2.22 - 2.18)	61.21 - 1.41 (1.45 - 1.41)
R_{pim} (I)	0.030 (1.665)	0.031 (0.899)	0.141 (1.171)	0.021 (1.022)
$CC_{1/2}$	0.999 (0.348)	0.999 (0.313)	0.987 (0.327)	0.999 (0.376)
I/σ (I)	13.5 (0.4)	12.8 (0.9)	5.1 (0.6)	18.3 (0.8)
Completeness (%)	99.9 (99.7)	100 (100)	98.7 (98.1)	99.6 (98.9)
Redundancy	40.1 (38.9)	38.7 (39.1)	14.1 (14.6)	38.8 (36.1)
**Refinement**				
Resolution (Å)	48.69 - 1.44 (1.49 - 1.44)	64.03 – 1.56 (1.62 – 1.56)	52.66 - 2.18 (2.26 - 2.18)	48.67 - 1.41 (1.46 - 1.41)
No. reflections	127,598	101,080	37,053	135,124
R_{work} / R_{free}	0.168 / 0.186	0.164 / 0.184	0.196 / 0.243	0.158 / 0.170
No. atoms				
Protein	4104	4052	3947	4141
Solvent	568	438	245	563
Ligand	-	60	42	76
B-factors (Å^2)				
Protein	25.3	29.4	40.3	27.5
Solvent	38.4	39.6	44.2	40.3
Ligand	-	33.6	44.4	37.4
RMS deviations				
Bond angles (°)	0.79	0.86	0.84	0.84
Bond lengths (Å)	0.0057	0.0064	0.0073	0.0062
Ramachandran (%)				
Outliers	0	0	0	0
Favoured	97.03	97.49	96.84	97.28

Table S1C: OXA-405 X-ray data collection and structure refinement statistics. *[a]Experiments were performed at [a]I03, [b]I04, [c]I24 beamlines of the Diamond Light Source (Didcot, UK), or [d]PROXIMA 2A beamline of SOLEIL (Paris, France). Outer shell statistics are brackets.*

	**Uncomplexed OXA-405[b]**	**OXA-405: Avibactam[b]**	**OXA-405: nacubactam[a]**
**PDB ID**	9H16	9H17	9H18
**Data collection**			
Space group	$P6_522$	$P6_522$	$P6_522$
Molecules/ASU	2	2	2
Cell dimensions			
a, b, c (Å)	122.08, 122.08, 159.12	122.30, 122.30, 160.18	123.69, 123.69, 159.35
α, β, γ (°)	90, 90, 120	90, 90, 120	90, 90, 120
Wavelength (Å)	0.9795	0.9537	0.9763
Resolution (Å)	63.57 - 1.56 (1.59 - 1.56)	57.13 - 1.46 (1.49 - 1.46)	57.66 - 1.33 (1.35 - 1.33)
R_{pim} (I)	0.053 (1.629)	0.030 (0.818)	0.027 (1.319)
$CC_{1/2}$	0.997 (0.348)	0.999 (0.286)	0.999 (0.297)
I/σ (I)	8.0 (0.5)	12.0 (0.5)	8.9 (0.1)
Completeness (%)	100 (100)	100 (100)	100 (98.1)
Redundancy	13.4 (13.3)	41.8 (42.2)	39.9 (40.5)
**Refinement**			
Resolution (Å)	61.04 - 1.56 (1.62 - 1.56)	57.13 - 1.46 (1.51 - 1.46)	57.66 - 1.33 (1.38 - 1.33)
No. reflections	99,172	121,931	138,682
R_{work} / R_{free}	0.175 / 0.193	0.174 / 0.196	0.183 / 0.199
No. atoms			
Protein	4025	4003	4010
Solvent	575	464	595
Ligand	-	60	76
B-factors (Å^2)			
Protein	27.7	25.9	28.0
Solvent	39.7	38.1	35.8
Ligand	-	27.0	32.0
RMS deviations			
Bond angles (°)	0.84	0.82	0.81
Bond lengths (Å)	0.0064	0.0058	0.0058
Ramachandran (%)			
Outliers	0	0.21	0
Favoured	97.17	97.68	97.03

Table S2: Crystallisation and ligand soaking experiment summary. *Seeds were generated using Seed Bead kit and Crystal Crusher (Hampton Research). % values are given in v/v.*

Protein	Ligand	Ligand soak time	Ligand concentration	Cryoprotection	Crystallisation condition	Temp (°C)	Seed
OXA-48	Apoenzyme	-	-	-	0.1 M HEPES pH 7.5, 33% PEG 400	10	-
	Nacubactam	30 mins	5 mM	20% glycerol	0.1 M Tris pH 8.8, 50% PEG 400	10	-
OXA-163	Apoenzyme	-	-	-	0.1 M Tris pH 9.0, 32% PEG 550	19	OXA-163 crystals
	Nacubactam ($P2_12_12$)	4 hour	5 mM	20% glycerol	0.1 M Tris pH 8.8, 40% PEG 400	10	OXA-163 crystals
	Nacubactam ($P6_522$)	16 hour	2.5 mM	20% glycerol	0.1 M Tris pH 9.0, 32% PEG 550	19	OXA-163 crystals
	Avibactam	1 hour	15 mM	20% glycerol	0.1 M Tris pH 9.0, 32% PEG 550	19	OXA-163 crystals
OXA-405	Apoenzyme	-	-	-	0.1 M Tris pH 8.5, 28% PEG 550	19	OXA-405 crystals
	Nacubactam	2 hour	5 mM	-	0.1 M Tris pH 9.0, 20% PEG 400	10	-
	Avibactam	1 hour	100 mM	20% glycerol	0.1 M Tris pH 8.5, 28% PEG 550	19	OXA-405 crystals

Table S3: Carbamylated Lys73 (KCX73) fitting statistics. *Statistics were calculated by the PDB validation server. Values are associated with chain A / chain B of each enzyme complex.*

Structure	KCX73 occupancy	KCX73 RSCC
OXA-48:nacubactam	-	-
OXA-163:avibactam	0.28 / -	0.96 / -
OXA-163:nacubactam (4 hour soak)	-	-
OXA-163:nacubactam (16 hour soak)	0.28 / 0.31	0.95 / 0.95
OXA-405:avibactam	0.40 / 0.31	0.97 / 0.97
OXA-405:nacubactam	-	-

Table S4: DBO complex ligand fitting statistics. *Statistics were calculated by PDB validation server. Values are associated with chain A / chain B of each enzyme complex.*

	Intact carbamoyl-enzyme		Desulfated hydroxylamine carbamoyl-enzyme	
**Structure**	**Occupancy**	**RSCC**	**Occupancy**	**RSCC**
OXA-48:nacubactam	0.62, 0.38 / 1	0.97, 0.97 / 0.96	-	-
OXA-163:avibactam	0.52 / 0.50	0.92 / 0.87	0.48 / 0.50	0.89 / 0.89
OXA-163:nacubactam (4 hour soak)	1 / 1	0.94 / 0.95	-	-
OXA-163:nacubactam (16 hour soak)	0.48 / 0.49	0.88 / 0.84	0.52 / 0.51	0.87 / 0.85
OXA-405:avibactam	0.54 / 0.58	0.93 / 0.95	0.46 / 0.42	0.92 / 0.93
OXA-405:nacubactam	0.66 / 0.77	0.93 / 0.94	0.34 / 0.23	0.88 / 0.94

Table S5: Primer sequences used to create the OXA-163 and OXA-405 expression constructs.

Reaction	Forward primer	Reverse primer
pOPIN-F OXA-163 cloning	5’-AAGTTCTGTTTCAGGGCCCGAAAGAATGGCAGGAAAACAAGAGCTG-3’	5’-ATGGTCTAGAAAGCTTTACGGGATGATTTTCTCCTGTTTGAG-3’
pOPIN-F OXA-405 cloning	5’-AAGTTCTGTTTCAGGGCCCGAAAGAATGGCAGGAAAACAAATCCTG-3’	5’-ATGGTCTAGAAAGCTTTACGGGATGATCTTTTCCTGCTTCAAC-3’

Table S6: Steady state kinetic parameters for nitrocefin hydrolysis by OXA-48, OXA-163 and OXA-405.

Enzyme	K_M (μM)	SD[a]	k_{cat} (s^{-1})	SD[a]	k_{cat}/K_M (s^{-1} μM^{-1})	SD[a]
**OXA-48**	114.4	59.2	515.0	71.1	5.2	2.5
**OXA-163**	13.4	7.3	64.5	7.4	6.4	4.5
**OXA-405**	33.9	19.9	113.9	20.3	4.1	2.1

[a]SD = standard deviation

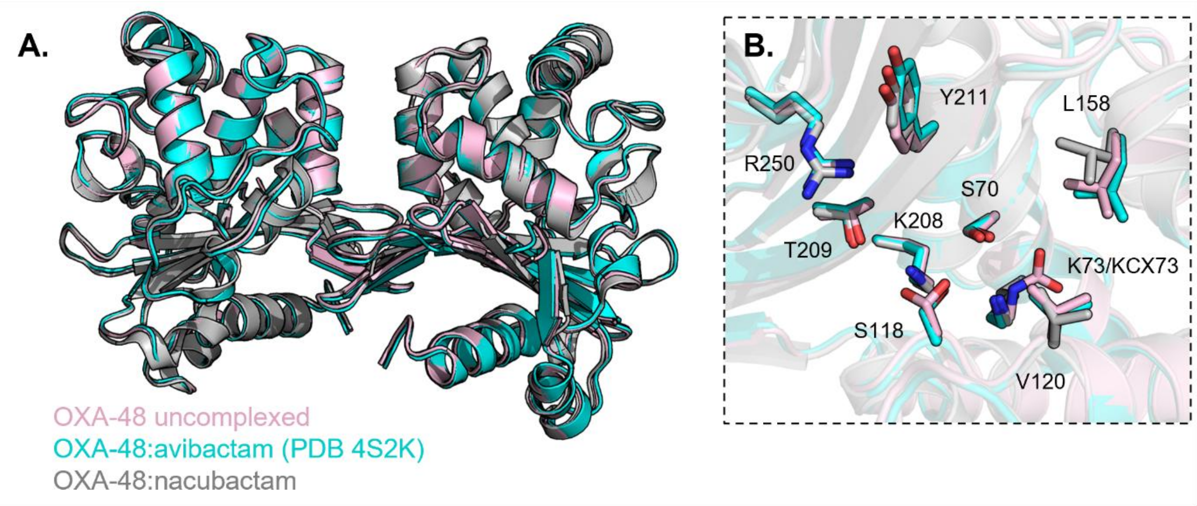

Figure S1: Overlay of uncomplexed and DBO-bound OXA-48 crystal structure views. *(A) Backbone structure with zoom in of one of the (B) active sites. RMSD values of nacubactam-bound OXA-48 when overlaid on uncomplexed and avibactam-bound OXA-48 (PDB 4S2K) are 0.41 Å and 0.53 Å, respectively. Active site residues are shown as sticks; the bound DBO inhibitors are not shown.*

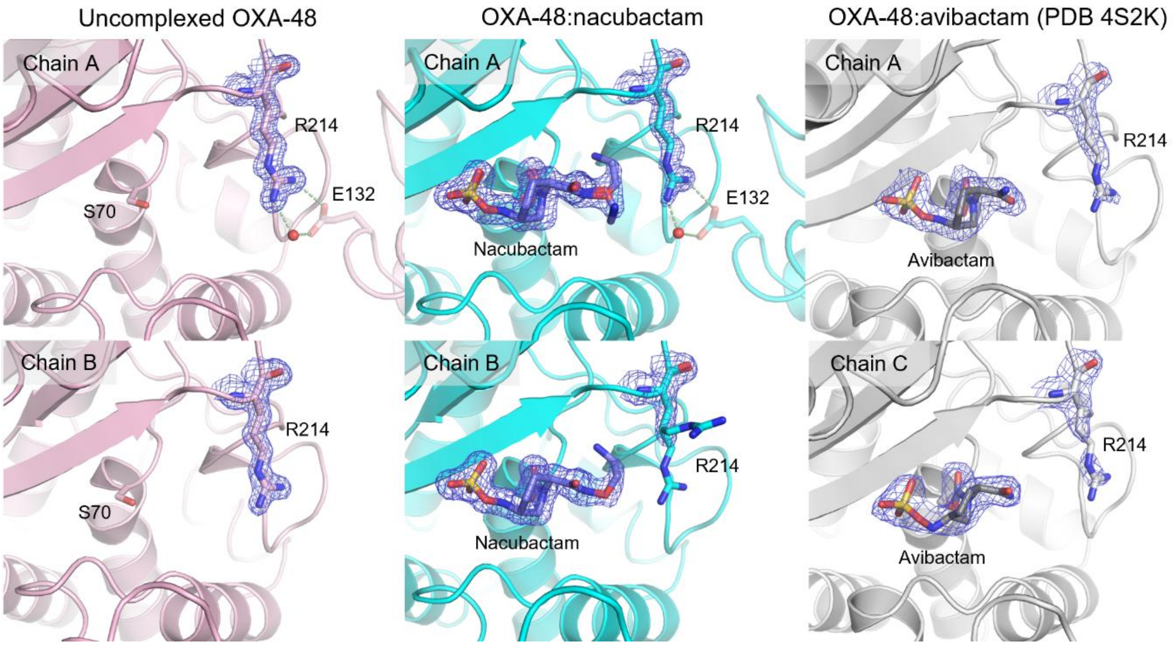

Figure S2: *In crystallo* conformations of Arg214 in uncomplexed and DBO-bound OXA-48. *Arg214 and DBO final 2F_o-F_c electron density, contoured to 1σ. Interacting crystal symmetry subunits not in the asymmetric unit are shown as transparent sticks/cartoon and Arg214-mediated crystal packing interactions highlighted by dashed lines, with bridging waters shown as red spheres. Each chain of the OXA-48 homodimer is labelled, with only one of the two homodimers (comprising chains A and C) in the crystallographic asymmetric unit of OXA-48:avibactam (PDB 4S2K) displayed.*

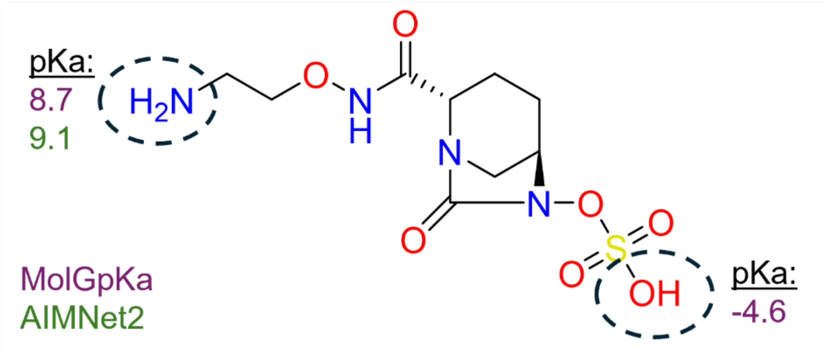

Figure S3: Predicted pKa values for the ionisable groups of Nacubactam. *MolGpKa[1] and AIMNet2[2] webserver pKa predictions are shown adjacent to the ionisable groups of nacubactam, which are highlighted by dashed circles.*

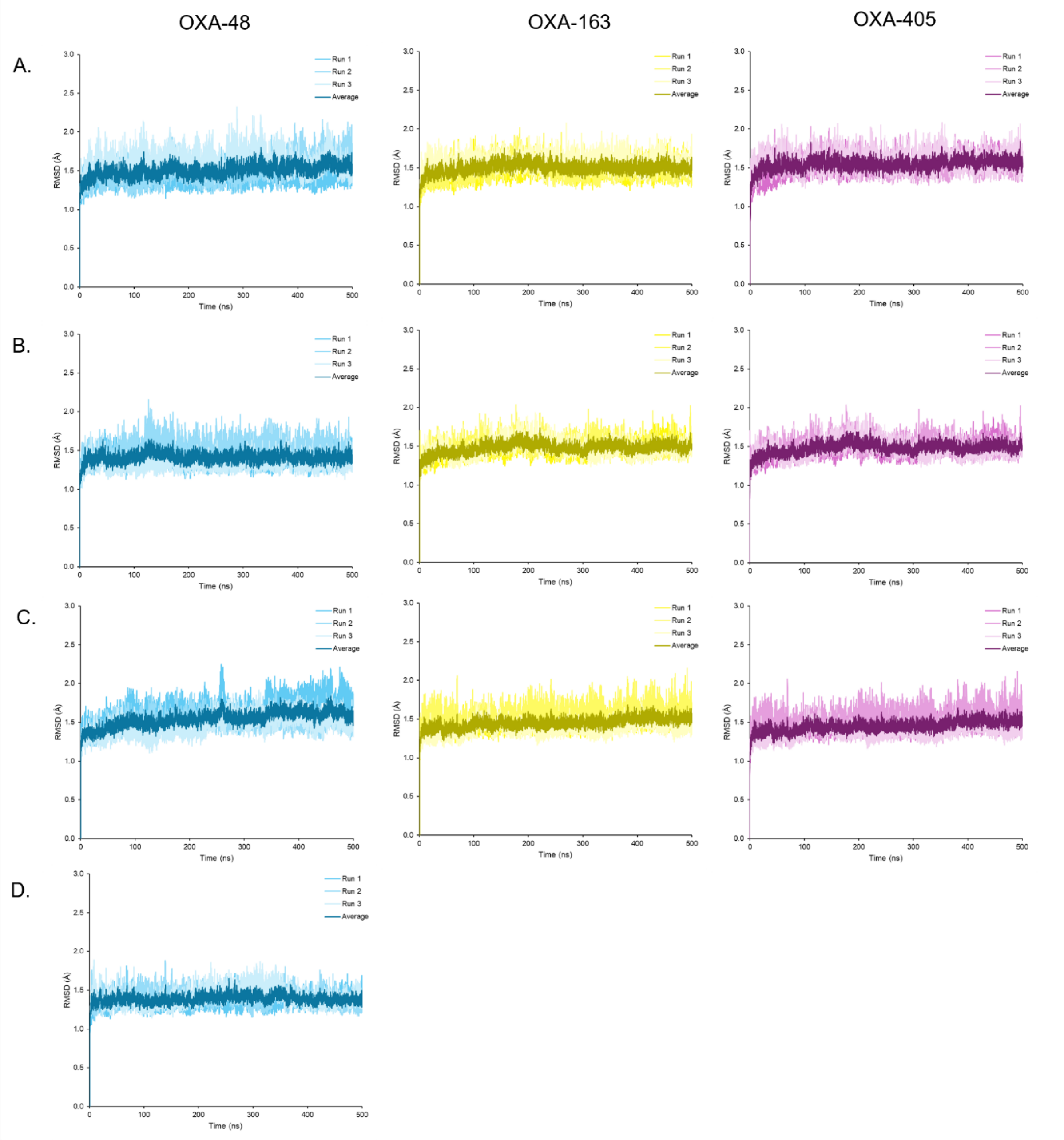

Figure S4: RMSD plots of MM MD simulation trajectories. *Simulations of (A.) uncomplexed, (B.) avibactam-bound, (C.) nacubactam-bound (C2 tail N protonated) and (D.) nacubactam-bound (C2 tail N deprotonated) OXA-48 (blue), OXA-163 (yellow), OXA-405 (pink) complexes. Each 500 ns simulation run is shown separately, with the average RMSD shown in the darkest shade of their respective colours.*

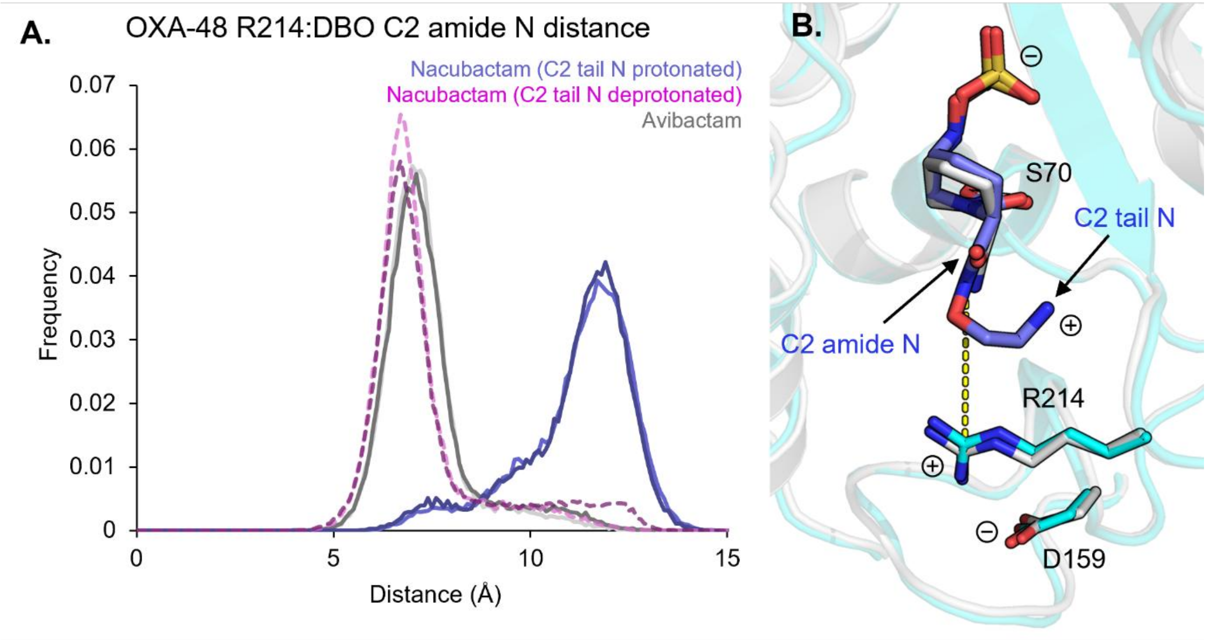

Figure S5: Distance analysis of Arg214 relative to avibactam and nacubactam across MD simulation trajectories. *(A) Frequency histogram of Arg214-C2 amide nitrogen distance over the OXA-48:DBO MD simulations. The darker shades of each colour correspond to the chain B active site complexes with OXA-48 and the lighter for chain A. (B) OXA-48 active site with avibactam and nacubactam overlayed, the distance between Arg214 and the DBO C2 amide nitrogen measured in panel A is labelled with a yellow dashed line.*

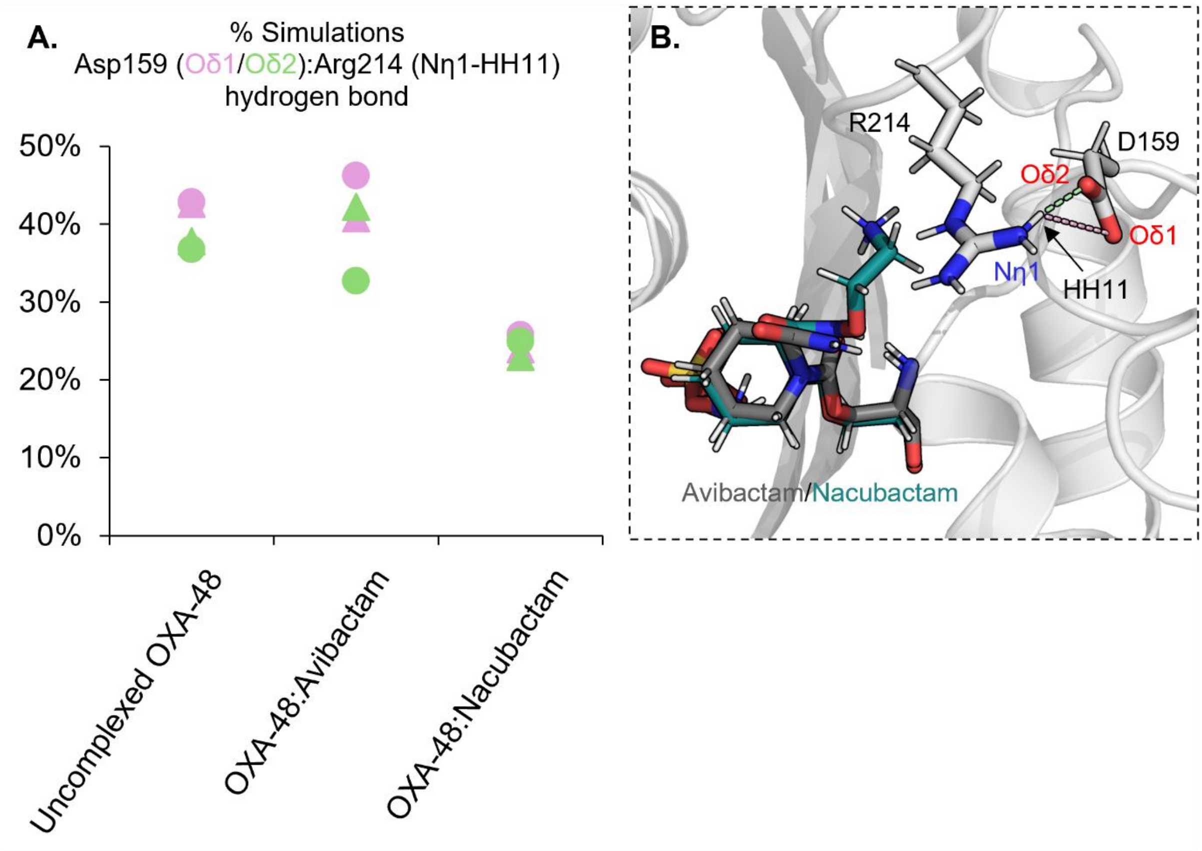

Figure S6: Hydrogen bonding analysis between Arg214 and Asp159 over MD simulation trajectories. *(A) Proportion (%) of DBO-bound and uncomplexed OXA-48 simulations where Arg214 (Nη1) and Asp159 (Oδ1, pink) or Asp159 (Oδ2, green) are involved in hydrogen bonding, with Nη1-HH11 being the shared hydrogen atom. 3 Å distance and 135° bond angle cutoffs were chosen for hydrogen bond assignment. Triangles represent chain A and circles chain B of OXA-48. (B) Overlay of energy minimised starting structures of OXA-48 bound to avibactam (grey) and nacubactam (blue) with the Asp159 (Oδ1):Arg214 (Nη1-HH11) and Asp159 (Oδ2):Arg214 (Nη1-HH11) interactions highlighted by pink and green dashed lines respectively.*

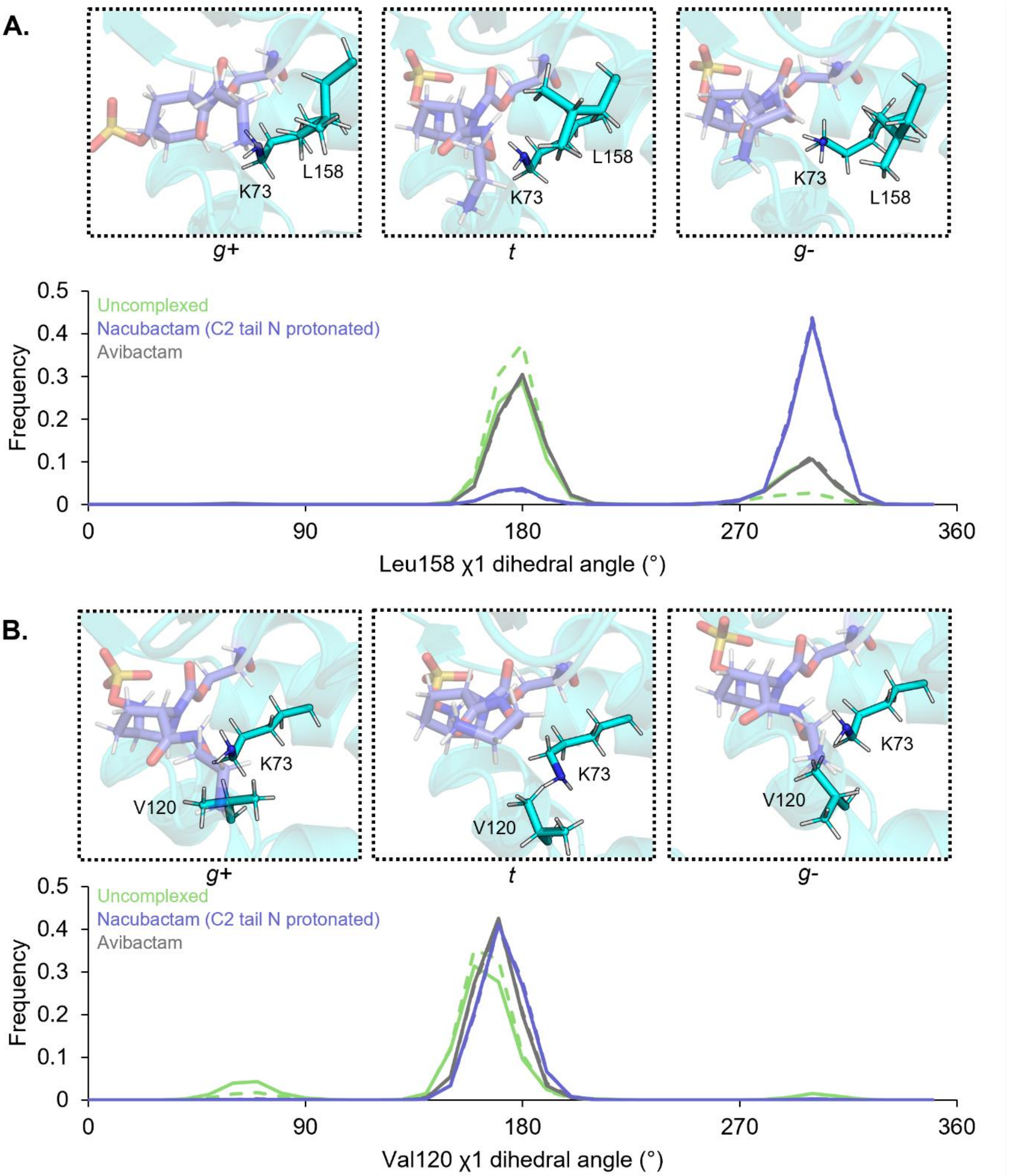

Figure S7: OXA-48 deacylating water channel conformational analysis over MD simulations. *(A) Leu158 χ_1 dihedral angle (N-Cα-Cβ-Cγ) and (B) Val120 χ_1 dihedral angle (N-Cα-Cβ-Cγ1) frequency histograms for simulation trajectories of nacubactam-bound (blue), avibactam-bound (grey) and uncomplexed (green) OXA-48. Representative simulation snapshots are shown above in the* g+ *(~60°),* t *(~180°) and* g- *(~300°) χ_1 rotamers of Leu158 and Val120. Lys73 and nacubactam carbamoyl-enzyme complex (blue, transparent) are also shown as sticks.*

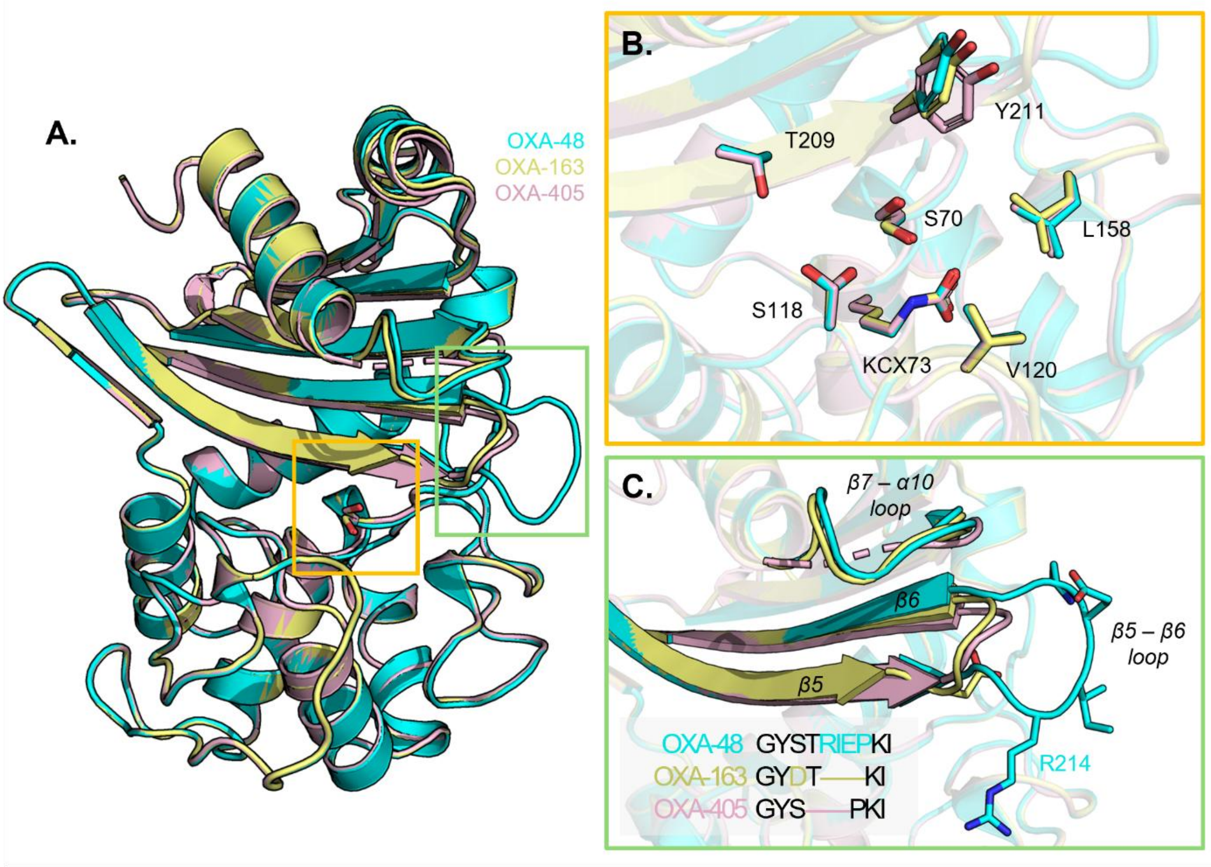

Figure S8: Overlay of OXA-48, OXA-163 and OXA-405 uncomplexed crystal structure views. *(A) Chain B overall topology, with zoom ins of the (B) active site and (C) β5 – β6 loops. RMSD values of the overlay are 0.12 Å and 0.14 Å for OXA-163 and OXA-405 apoenzymes respectively, relative to OXA-48. Key active site residues and substituted/deleted residues across OXA-48 variants are shown as sticks. Unmodelled residues are represented by a dashed line. β5 – β6 loop sequence overlay is shown in (C).*

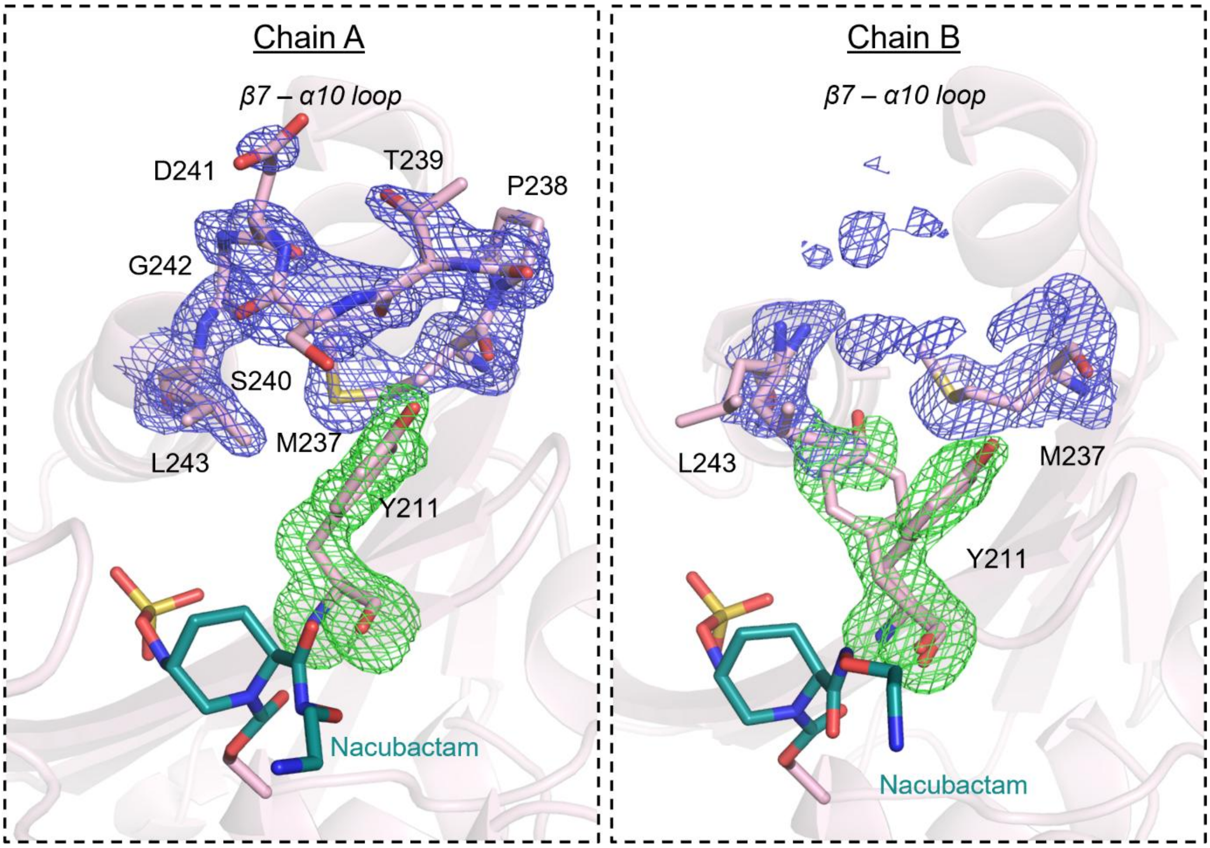

Figure S9: Conformational flexibility of Tyr211 and β7 – α10 loop in the OXA-405:nacubactam complex. *The Tyr211 unbiased* F_o-F_c *omit map is shown as a green mesh, contoured at 3σ. The β7 – α10 (residues 237-243) final* $2F_o$-F_c *electron density is represented as a blue mesh, contoured at 1σ. The chain B β7 – α10 map* $2F_o$-F_c *was generated using the chain A loop superimposed onto the chain B of OXA-405, contouring the electron density around the overlaid loop.*

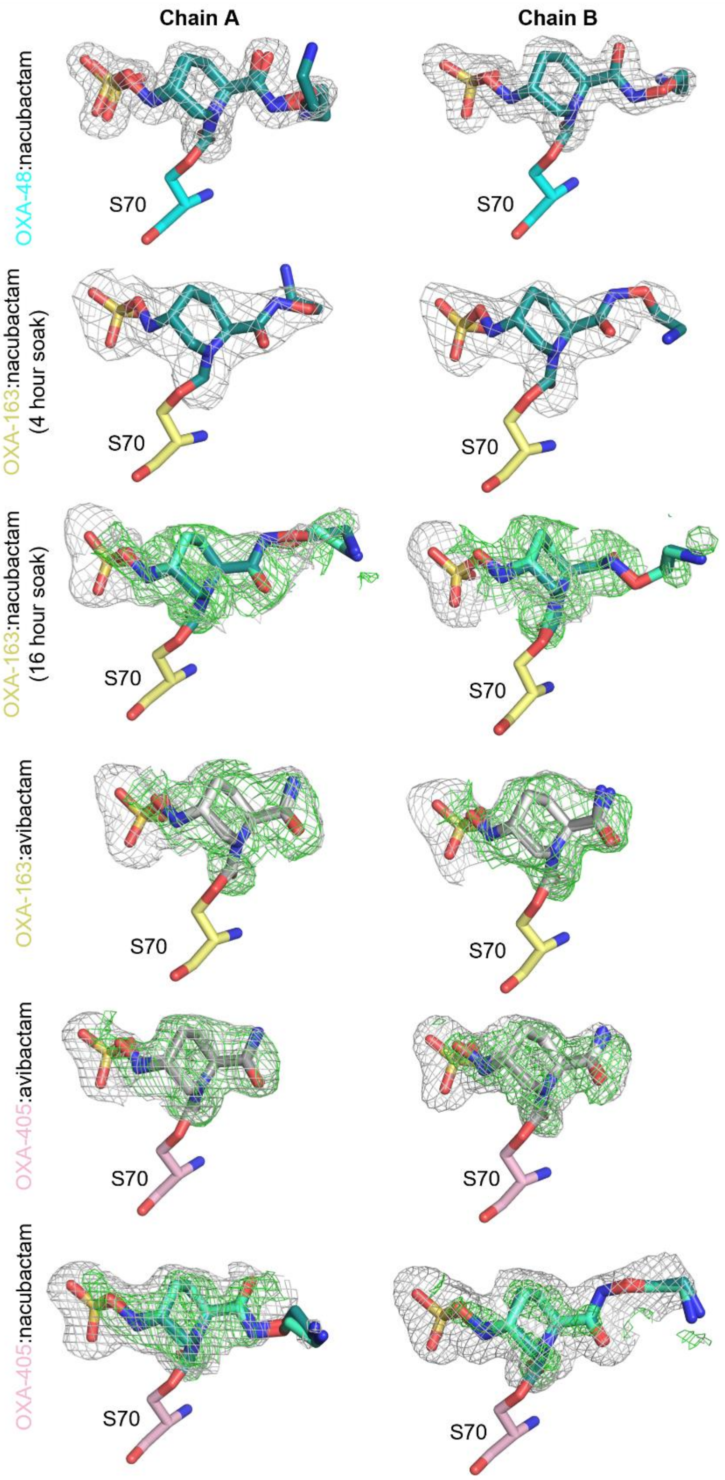

Figure S10: DBO-derived carbamoyl-enzyme unbiased F_o-F_c omit maps. *Maps are contoured at 3σ, with the grey mesh calculated following removal of intact carbamoyl-enzyme and the green mesh following removal of desulfated hydroxylamine carbamoyl-enzyme. DBO-Ser70 active site carbamoyl-enzyme complexes are shown as sticks, with desulfated DBO-derived carbamoyl-enzymes coloured in lighter shades of blue and grey for nacubactam and avibactam respectively.*

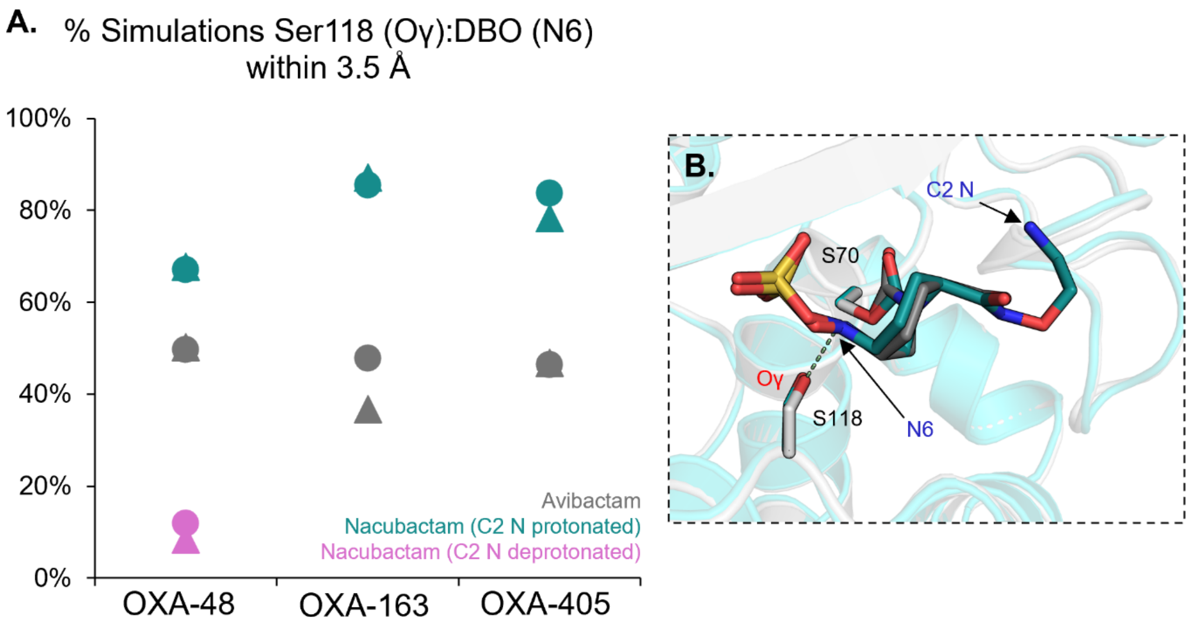

Figure S11: Analysis of distances between Ser118 Oγ and DBO N6 atoms in simulations of DBO carbamoyl complexes of OXA-48-related enzymes. *(A) Proportion (%) of simulations where Ser118 (Oγ) and N6 of the DBO-derived carbamoyl-enzyme complex are within 3.5 Å, for each OXA-48-like enzyme. Triangles represent chain A and circles chain B of individual DBO complexes. (B) Overlay of OXA-48 avibactam- (PDB 4S2K[3], grey) and nacubactam-bound (turquoise) structures, with the Ser118:DBO distance represented by a pale green dashed line.*

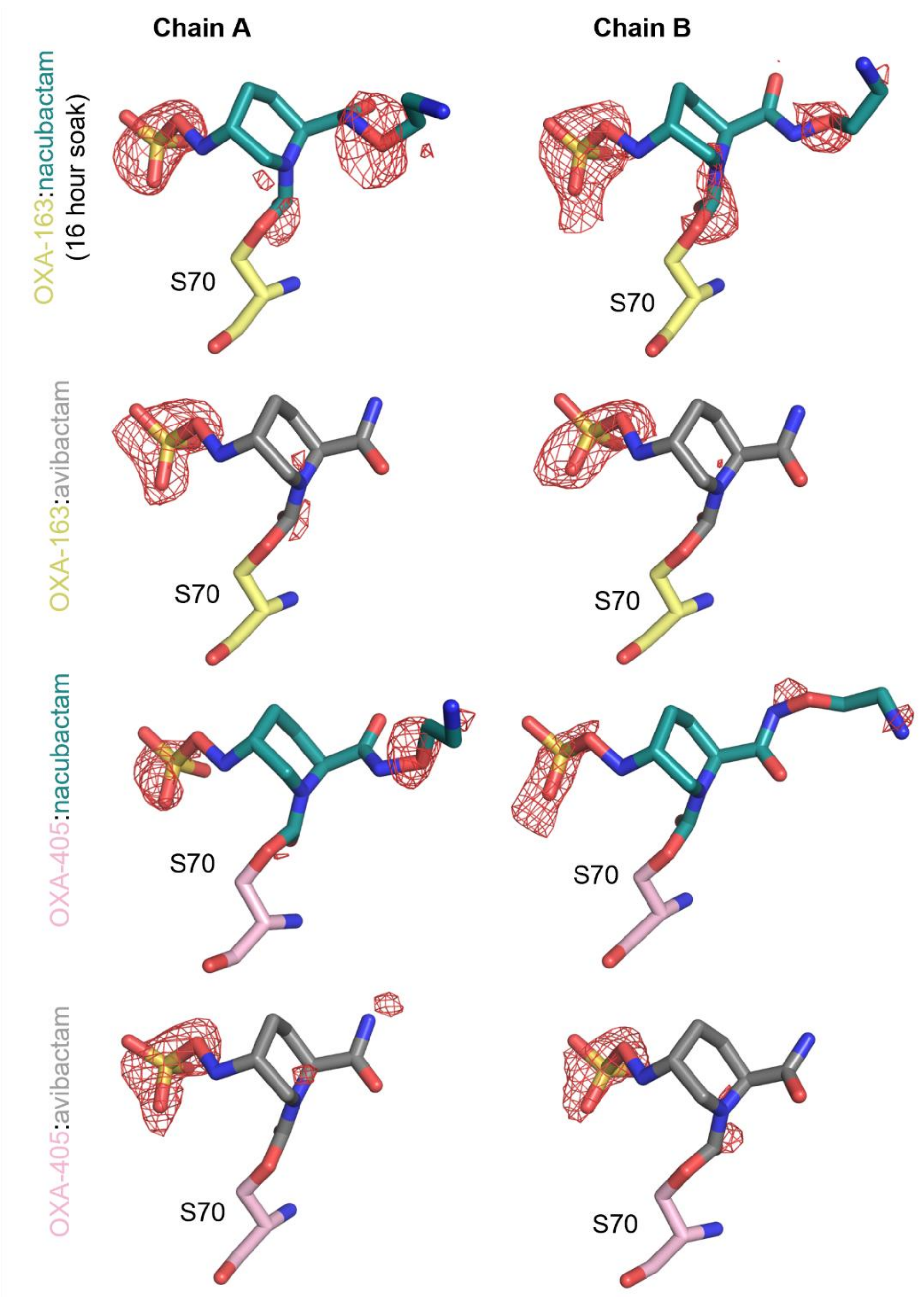

Figure S12: Intact DBO-derived carbamoyl-enzyme negative *F*o-*F*c difference maps. *Maps were calculated using refined models prior to the addition of the dual-occupancy desulfated carbamoyl-enzymes. Maps are contoured at -3σ and represented as a red mesh. DBO-Ser70 carbamoyl-enzyme complexes are shown as sticks.*

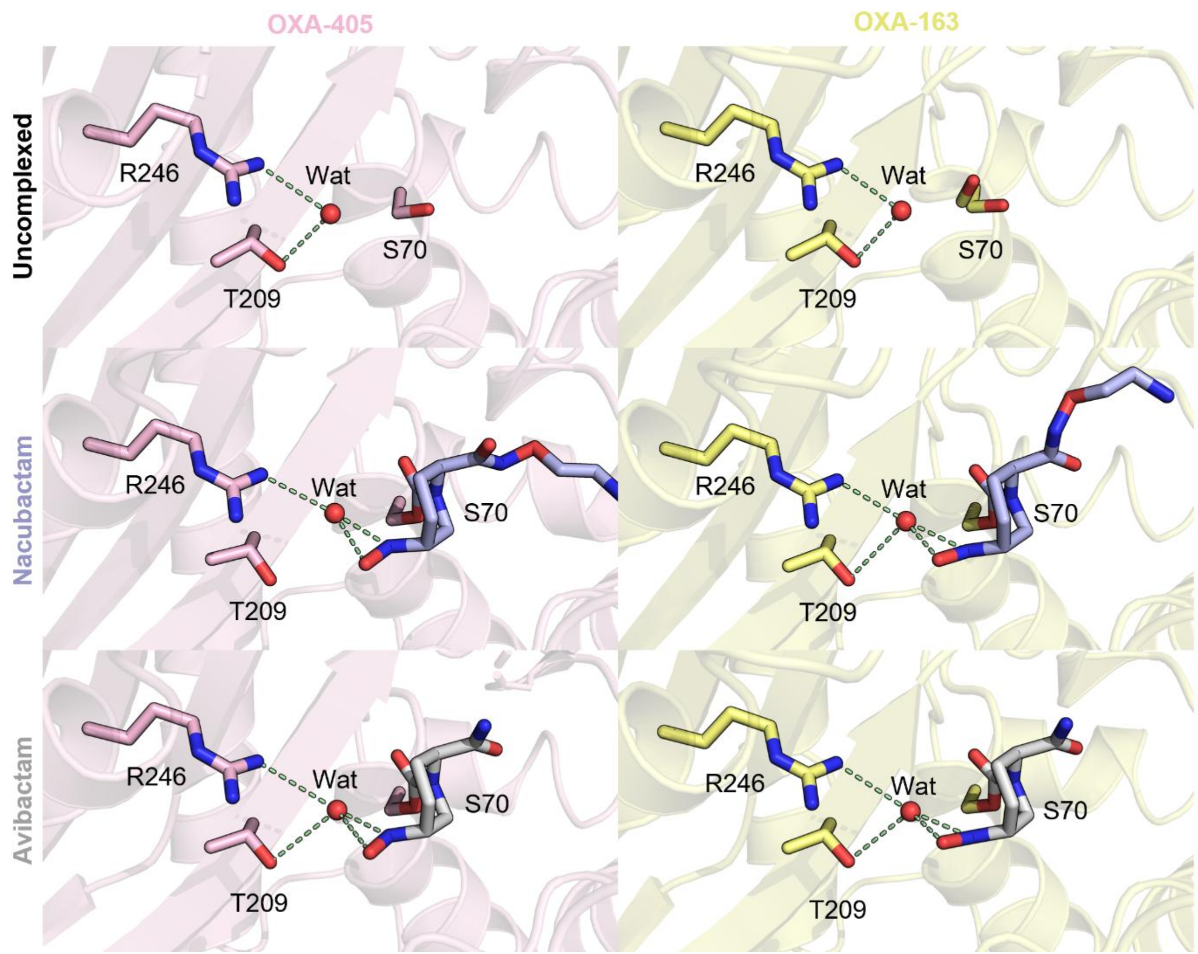

Figure S13: Water-mediated interactions between the hydroxylamine of desulfated DBO-derived complexes with OXA-405 and OXA-163 active sites. *Active site residues and DBO-derived carbamoyl-enzyme complexes are shown as sticks, waters as red spheres and possible hydrogen bonding interactions (within 3.4 Å) as dashed lines. Only chain A active sites are shown for each structure.*

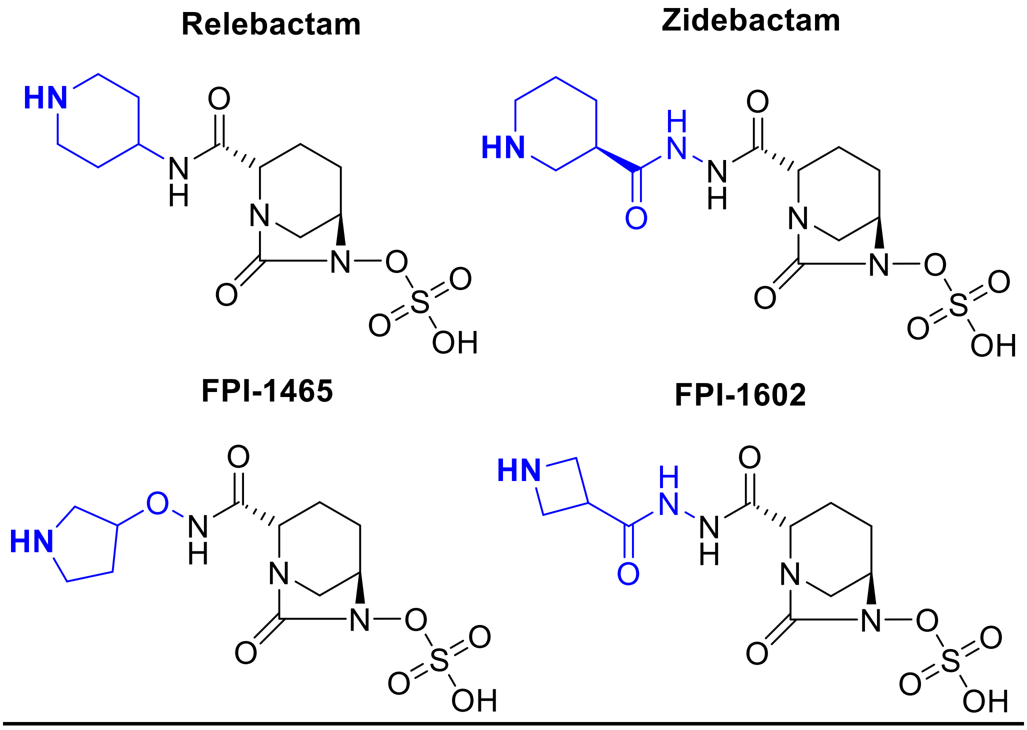

Figure S14: Alternative DBO inhibitors with ionisable C2 substituents. *Example DBO clinical, pre-clinical and investigational inhibitors that have modified C2 substituents (blue), compared to avibactam, containing an ionisable nitrogen (in bold).*

References

(1) Pan, X.; Wang, H.; Li, C.; Zhang, J. Z. H.; Ji, C. MolGpka: A Web Server for Small Molecule pKa Prediction Using a Graph-Convolutional Neural Network. *J. Chem. Inf. Model.* **2021**, *61* (7), 3159–3165. https://doi.org/10.1021/acs.jcim.1c00075.

(2) Anstine, D.; Zubatyuk, R.; Isayev, O. AIMNet2: A Neural Network Potential to Meet Your Neutral, Charged, Organic, and Elemental-Organic Needs. ChemRxiv October 12, 2023. https://doi.org/10.26434/chemrxiv-2023-296ch.

(3) King, D. T.; King, A. M.; Lal, S. M.; Wright, G. D.; Strynadka, N. C. J. Molecular Mechanism of Avibactam-Mediated β-Lactamase Inhibition. *ACS Infect. Dis.* **2015**, *1* (4), 175–184. https://doi.org/10.1021/acsinfecdis.5b00007.
